# Association between host wing morphology polymorphism and *Wolbachia* infection in *Vollenhovia emeryi* (Hymenoptera: Myrmicinae)

**DOI:** 10.1002/ece3.6582

**Published:** 2020-07-28

**Authors:** Pureum Noh, Seung‐Yoon Oh, Soyeon Park, Taesung Kwon, Yonghwan Kim, Jae Chun Choe, Gilsang Jeong

**Affiliations:** ^1^ Division of EcoScience Ewha Womans University Seoul Korea; ^2^ National Institute of Ecology Seochun‐gun Korea; ^3^ School of Biological Sciences Seoul National University Seoul Korea; ^4^ Interdisciplinary Program of EcoCreative The Graduate School Ewha Womans University Seoul Korea; ^5^ Division of Forest Ecology Korea Forest Research Institute Seoul Korea; ^6^ Department of Physics Konkuk University Seoul Korea; ^7^ Present address: Herbal Medicine Resources Research Center Korea Institute of Oriental Medicine Naju Korea

**Keywords:** divergence, population structure, *Vollenhovia emeryi*, wing polymorphism, *Wolbachia* infection

## Abstract

Many eusocial insects, including ants, show complex colony structures, distributions, and reproductive strategies. In the ant *Vollenhovia emeryi* Wheeler (Hymenoptera: Myrmicinae), queens and males are produced clonally, while sterile workers arise sexually, unlike other ant species and Hymenopteran insects in general. Furthermore, there is a wing length polymorphism in the queen caste. Despite its evolutionary remarkable traits, little is known about the population structure of this ant species, which may provide insight into its unique reproductive mode and polymorphic traits. We performed in‐depth analyses of ant populations from Korea, Japan, and North America using three mitochondrial genes (COI, COII, and Cytb). The long‐winged (L) morph is predominant in Korean populations, and the short‐winged (S) morph is very rare. Interestingly, all L morphs were infected with *Wolbachia*, while all Korean S morphs lacked *Wolbachia*, demonstrating a association between a symbiont and a phenotypic trait. A phylogenetic analysis revealed that the S morph is derived from the L morph. We propose that the S morph is associated with potential resistance to *Wolbachia* infection and that *Wolbachia* infection does not influence clonal reproduction (as is the case in other ant species).

## Graphical abstract

The figure clearly shows the wing morphology and Wolbachia infection status.

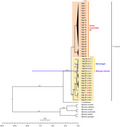

## 1 INTRODUCTION

Population structure analyses using genetic data provide extensive information about populations, including genetic distribution, genetic diversity, gene flow, and selection. Furthermore, these analyses can be used to evaluate relationships between secondary traits such as phenotype, reproductive strategy, and symbiotic bacterial communities. Among secondary traits, wing morph is the principal phenotype associated with direct dispersal, distribution, and reproductive strategies in insects (Ikeda, Nishikawa, & Sota, 2012; Lin, Yao, Wang, Emlen, & Lavine, 2016; McCulloch et al., 2019; Roff, 1986). In ants, wings play a salient role in nuptial flight, which determines dispersal and breeding success. However, the wing is not a *sine qua non* in several species. Winglessness and wing reduction in reproductive ants are widespread across all subfamilies (Buschinger & Heinze, 1992; Heinze & Tsuji, 1995; Peeters, 1991, 2012; Peeters & Ito, 2001; Tinaut & Heinze, 1992; Villet, 1991).

*Vollenhovia emeryi* Wheeler (Hymenoptera: Myrmicinae) is a common ant species endemic to East Asia; this species has invaded North America (Kjar & Suman, 2007; Wetterer, Guenard, & Booher, 2015; Wright & Kubik, 2011). It is polymorphic for normal long and aberrant short wing length in queens. The two morphs are not thought to coexist in nature, and colonies of the long‐winged (L) morph are typically monogynous, while short‐winged (S) morph colonies are polygynous (Kinomura & Yamauchi, 1994). Unlike other ant species and Hymenopteran insects in general, queens and males are produced clonally, while sterile workers arise sexually (Kobayashi, Hasegawa, & Ohkawara, 2008, 2011; Ohkawara, Nakayama, Satoh, Trindl, & Heinze, 2006). This unusual clonal reproduction system is very similar to the system first found in some populations of the little fire ant *Wasmannia auropunctata* (Foucaud, Estoup, Loiseau, Rey, & Orivel, 2010; Foucaud et al., 2006, 2007; Fournier et al., 2005) and in the highly invasive longhorn crazy ant *Paratrechina longicornis* (Pearcy, Goodisman, & Keller, 2011). Selfish clonal reproduction in both sexes might evolve without allowing genetic contamination by the opposite sex, thereby giving rise to genetically homogeneous clonal lineages, despite the cost for abandoning genetic diversity and thus the ability to tolerate environmental changes (Fournier et al., 2005; Matsuura, 2010; Pigneur, Hedtke, Etoundi, & Van Doninck, 2012). In some other hymenopteran insects, reproductive manipulators such as *Wolbachia* cause host's clonal reproduction (Jeong & Stouthamer, 2004; Pannebakker, Pijnacker, Zwaan, & Beukeboom, 2004).

The *Wolbachia* bacterium is a maternally‐inherited endosymbiont that infects a wide variety of invertebrates such as insects (including ants) and other arthropods (Bourtzis & Miller, 2008; Correa & Ballard, 2016; Hilgenboecker, Hammerstein, Schlattmann, Telschow, & Werren, 2008; Kautz, Rubin, Russell, & Moreau, 2013; Werren, 1997; Zientz, Feldhaar, Stoll, & Gross, 2005; Zug & Hammerstein, 2015). Infection induces various types of reproductive alterations in the host, including cytoplasmic incompatibility, feminization, male‐killing, and parthenogenesis (Fujii, Kubo, Ishikawa, & Sasaki, 2004; Jeong & Suh, 2008; Stouthamer, Breeuwer, & Hurst, 1999). The ants are attractive taxa as the host of *Wolbachia* due to their eusocial haplodiploids with generally female‐biased sex ratios (Russell, 2012). Approximately 30% of ant species have been estimated to be facultatively infected with *Wolbachia* (Russell, 2012; Russell et al., 2012). Recent studies reported the evidence for *Wolbachia*‐associated sex‐, caste‐ratio, and colony life cycle changing in ants (de Bekker, Will, Das, & Adams, 2018; Pontieri, Schmidt, Singh, Pedersen, & Linksvayer, 2017; Singh & Linksvayer, 2020; Wenseleers, Sundström, & Billen, 2002). However, the effect of *Wolbachia* infection is still poorly understood in their host ants.

The present study focused on the association between the ant species, *V. emeryi* with unusual clonal reproduction system, and Wolbachia. The specific aims of this study were to examine (a) the population genetic structure of the mitochondrial genes of *V. emeryi*; (b) the phylogeographic relationships among the two winged morphs from Korea and Japan; (c) the approximate divergence time of the two winged morphs; (d) the ubiquity of Wolbachia infection in this ant species; and (e) potential relationships between host phenotype and Wolbachia infection.

## 2 MATERIALS AND METHODS

### 2.1 Collection of *V. emeryi* samples

Either individuals or colonies of *V. emeryi* were collected from 74 locations between 2010–2013, including 65 locations in South Korea, eight in Japan, and one in the United States (Table S1). Since this ant species usually lives in moist conditions, particularly in rotten wood, the collection sites were mountains or forests, and a large number of sampled colonies were found under the bark of rotten trees. For genetic analyses, 1–3 individuals per colony were sacrificed in 100% EtOH.

### 2.2 DNA extraction and PCR

Genomic DNA was extracted from the whole body of *V. emeryi* samples stored in 100% EtOH using a commercial kit (Qiagen DNeasy Blood and Tissue Kit, Hilden, Germany), according to the manufacturer's instructions. The extracted genomic DNA was kept at −20°C until further analyses.

Kobayashi, Hasegawa, and Ohkawara (2008), Kobayashi, Hasegawa, and Ohkawara (2011) revealed that the nuclear genetic relationships among sexual forms of two wing morphs (L queen, L male, S queen, and S male) are different from mitochondrial genetic relationships. The S queen was distinguished from other morphs by its nuclear genetic similarity, while the four sexual forms were divided into two groups according to wing type based on mitochondrial genetic similarity (Kobayashi et al., 2008, 2011). Therefore, mitochondrial genes were chosen to investigate the relationship between the L morph and the S morph.

The primer sets used for analyses are listed in Table 1. The primer sets targeting the three mitochondrial genes were specific for *V. emeryi* (exceptions were Pat for COI and 21v2 and r8v2 for COII). The PCR temperature profile was as follows: denaturation for 3 min at 95°C, followed by 35 cycles of 1 min at 95°C, 1 min at each annealing temperature, 1–2 min at 72°C, and a final extension step at 72°C for 5 min. To examine the infection status of *Wolbachia*, diagnostic PCR was performed using the *Wolbachia*‐specific primer set WspecF, R (Werren & Windsor, 2000) at the appropriate annealing temperature (Table 1). To confirm *Wolbachia* infection, PCR using a fragment of the cell cycle gene *FtsZ* was performed using samples that presented as *Wolbachia*‐free in the first diagnostic PCR with a positive control. *Wolbachia*‐specific *FtsZ* primers were used for PCR according to the method described by Baldo et al. (2006). The Maxime PCR PreMix Kit (iNtRON Biotechnology, Seongnam, Korea) was used for each amplification along with 16 µl of distilled water, 1 µl of each primer (10 pmol), and 5 ng of template DNA.

**TABLE 1:** Primers used in this study

Locus	Primer name	Primer sequences (5′−3′)	Purpose	Annealing temperature (°C)	Reference
COI	F: LCO‐a R: HCO‐a	CCYCGWATAAATAAYATAAGATTTTGA TAAACTTDGGRTGWCCAAAAAATCA	PCR and Sequencing	45–50	Designed to be specific for *V. emeryi*
	F: Engel R: Pat	GAGGAGGAGACCCCATTTTAT TCCAATGCACTAATCTGCCATATTA	PCR and Sequencing	45–50	Designed to be specific for *V. emeryi* Simon et al. (1994)
COII	F: 21v2 R: r8v2	ATATTCACAATTGGGTTAGATGTAGA AGCTGCGGCTTCAAATCCA	PCR	55–60	Kobayashi, Tamura, Okamoto, Hasegawa, and Ohkawara, (2012)
	F: Ve13‐sF1 R: Ve13‐sR1 F: Ve13‐sF2 R: Ve13‐sR2	ATGTACATTATTTGGGAAGCACTAGC AATGTCAAATTATTTATTGGGATAGGG ATTAACCGCCCTGGAATATTTTT TTGTTAGAGATAGGGGGACACAA	Sequencing	—	Designed to be specific for *V. emeryi*
Cytb	F: VeCB‐F1 R: VeCB‐R1	TGCCCTAATACTCAATTAGCCTTT TGTATGGGGATTCAATTACTTGTG	PCR and Sequencing	52–60	Designed to be specific for *V. emeryi*
16s rRNA	F: WspecF R: WspecR	CATACCTATTCGAAGGGATAG AGCTTCGAGTGAAACCAATTC	*Wolbachia*‐specific diagnostic PCR	55	Werren and Windsor (2000)
*ftsZ*	F: ftsZ_F1 R: ftsZ_R1	ATYATGGARCATATAAARGATAG TCRAGYAATGGATTRGATAT	*Wolbachia*‐specific diagnostic PCR	54	Baldo et al. (2006)

Abbreviations: F, forward primer; R, reverse primer.

PCR amplification was conducted using either a PTC‐100 Programmable Thermal Controller (MJ Research, Inc.) or a PeqSTAR Universal Gradient Thermocycler (Peqlab Gmbh). The PCR amplicons were visualized in a 1% agarose gel dyed with TopGreen Nucleic Acid Gel Stain (Genomic Base) and purified using a commercial kit (QIAquick PCR Purification Kit; Qiagen) prior to sequencing. In all cases, sequences were read in both directions for maximum clarity.

### 2.3 Data analysis

#### 2.3.1 Population genetic structure and demographic analyses

The resultant sequences were aligned and analyzed using ClustalW embedded in MEGA (ver. 5.2; Kumar, Nei, Dudley, & Tamura, 2008; Kumar, Tamura, & Nei, 1994; Thompson, Higgins, & Gibson, 1994). The aligned sequences were submitted to GenBank along with the translated amino acid sequences. GenBank accession numbers are shown in Table S1. Haplotypes were determined using DnaSP (ver. 5.10; Librado & Rozas, 2009).

A good correlation has been reported between ground vegetation and ant community diversity (Andersen, 1995, 1997; Lubertazzi & Tschinkel, 2003). Hence, sequence data were grouped according to regions on a vegetation map of the Korean peninsula overlaid with isothermal lines (Yi, 2011). Within the range of deciduous broad‐leaved forests (temperate zone), the central area was designated region A, the southwestern area was designated region B, and the southeastern area was designated region C. Region D represented the evergreen broad‐leaved forest (subtropical‐warm temperate zone), and region E represented Yeosu‐si, a central spot on the southern coast, based on the unique characteristics of the sample collected at this site. Jeju Island, a volcanic island far from the mainland of Korea, was labeled region F. Regions G and H were the USA and Japan, respectively.

Molecular diversity indices were calculated for all eight regions and each gene. Analysis of molecular variance (AMOVA) among regions, including the overall fixation index statistics (*F*_ST_) and pairwise *F*_ST_, was performed with 1,000 permutations. To test the model of evolution and demographic expansion for the COI gene, neutrality tests (Tajima's *D* and Fu's *F*_S_; Fu, 1997; Tajima, 1989) and mismatch distribution tests were performed with 1,000 replicates using Arlequin (ver. 3.5.1.2; Excoffier & Lischer, 2010). Based on the mismatch distribution, demographic expansion patterns for seven regions (excluding region G, i.e., the USA, which lacks variation) were determined using DnaSP and edited using Microsoft PowerPoint 2013.

Genetic distances among haplotypes were calculated after selecting the best‐fit substitution model in MEGA. The median‐joining algorithm was employed to infer phylogenetic relationships among the haplotypes using Network (ver. 4.6.10), with a fixed connection limit at 1,000 steps between haplotypes (Bandelt, Forster, & Röhl, 1999). The haplotype network was edited manually and reconstructed with the regional distribution data using Adobe Illustrator CS6 (Adobe Inc.).

#### 2.3.2 Estimation of the origin of the S morph lineage

The relaxed clock method was used to estimate the approximate divergence date of the S gyne from the L queen. Three sets of monophyletic lineages, thought to have diverged approximately 1 MYA (million years ago), 2 MYA, and 3 MYA, were used, that is, *Myrmica excelsa* and *M. taediosa*, *M. sulcinodis* and *M. xavieri*, and *M. tobiasi* and *M. georgica*, respectively (GenBank Accession No: FJ824432, GQ255131, GQ255141, GQ255197, GQ255192, and GQ255145; Jansen & Savolainen, 2010). Jansen and Savolainen (2010) estimated the divergence time of holarctic *Myrmica* ants using mitochondrial and nuclear genes; the COI data and their estimates were extracted.

The HKY + G + I model (gamma distribution shape value: 1.26247; proportion of invariant sites: 0.61287) was selected as the best fit evolutionary substitution model based on the Bayesian information criterion, as determined using MEGA (Kumar et al., 1994, 2008). For the clock method, Bayesian Markov chain Monte Carlo was run for 100 million generations. Trees were sampled every 1,000 generations using BEAST (ver. 1.8.0; Drummond & Rambaut, 2007). Posterior distributions for parameter estimates and likelihood scores were visualized using Tracer (ver. 1.5) to examine tree appropriateness. The trees were consolidated to a maximum clade credibility tree with median heights after discarding the first 15,000 trees as burn‐in. The resultant tree was visualized, with 95% HPD (highest posterior density), using FigTree (ver. 1.40). It was further edited with additional data using Adobe Illustrator CS6 (Adobe Inc.).

#### 2.3.3 Association between wing morphology and *Wolbachia* infection status

The chi‐square independence test in SPSS (Release 17.0) was used to examine whether there is a relationship between wing morph and *Wolbachia* infection. For statistical analysis, the three USA individuals were excluded owing to uncertainty with respect to their wing morphology.

## 3 RESULTS

### 3.1 Molecular diversity

We analyzed the mitochondrial COI (1,224 bp), COII (663 bp), and Cytb (839 bp) genes for a specimen from each of the 145 ant colonies. We identified 37 (COI), 25 (COII), and 26 (Cytb) unique haplotypes (Table S2). Overall molecular diversity indices for eight regions and for each gene are shown in Table 2 (COI) and Table S3 (COII and Cytb). Both nucleotide diversity (π) and haplotype diversity (*h*) decreased in the order COI (π: 0.086 ± 0.078; *h*: 0.557 ± 0.289), Cytb (π: 0.078 ± 0.090; *h*: 0.455 ± 0.233), and COII (π: 0.062 ± 0.062; *h*: 0.430 ± 0.278) and were highest in region *F* (Jeju island; π: 0.233, *h*: 0.867 for COI; π: 0.261, *h*: 0.733 for Cytb; π: 0.202, *h*: 0.733 for COII).

**TABLE 2:** Molecular diversity indices for eight regions of mitochondrial COI

Index	Region (*N*_s_)								Total (145)
	A (36)	B (14)	C (24)	D (17)	E (23)	*F* (6)	G (3)	H (22)	
*N*_h_	12	3	5	9	4	4	1	7	37
*nTi*/*nTv*	12.2	24.5	47	13.5	—	10.5	—	17	20.783 ± 13.769
π	0.063	0.176	0.040	0.069	0.043	0.233	0	0.061	0.086 ± 0.078
*h*	0.560	0.473	0.377	0.853	0.549	0.867	0	0.779	0.557 ± 0.289

Abbreviations: *h*, haplotype diversity; *N*_h_, number of haplotypes; *N*_s_, number of samples examined; *nTi*/*nTv*, the ratio of transitions to transversions; π, nucleotide diversity.

### 3.2 Population genetic structure and demographic analyses

The observed *F*_ST_ values for COI, COII, and Cytb were 0.781, 0.687, and 0.803, respectively, indicating that the regional populations are genetically isolated. For COI, the estimated migration rate (*N*_e_ *m,* where *N*_e_ is the effective population size and *m* is the proportion of the population that migrates in each generation) was 0.07 migrants per generation (Slatkin, 1987; Slatkin & Barton, 1989). All three genes showed greater variation among regions (73.25%–75.90%) than within regions (0%–4.37%; Table 3; Table S4). We detected high *F*_ST_ in pairwise combinations between regions E, F, G, and H (Table 4; Tables S5 and S6). For the COI gene, 23 out of 28 pairwise combinations showed significant differentiation, and the highest pairwise *F*_ST_ was 0.91731 for the comparison between region C and region G (Table 4).

**TABLE 3:** AMOVA for mitochondrial COI of *V. emeryi*

Source of variation	*df*	Percentage of variation
Among regions	7	74.81
Among populations within regions	53	3.27
Within populations	84	21.92
Total	144	100.00

Abbreviation: *df*, degrees of freedom.

**TABLE 4:** Population pairwise *F*_ST_ values between regions for COI

	Region A	Region B	Region C	Region D	Region E	Region F	Region G	Region H
Region A	—							
Region B	0.09915*	—						
Region C	−0.01541	0.09881*	—					
Region D	−0.00838	0.04035	−0.01882	—				
Region E	0.88699**	0.75978**	0.91318**	0.88401**	—			
Region F	0.76830**	0.50484**	0.80042**	0.72167**	0.69558**	—		
Region G	0.87031**	0.64864**	0.91731**	0.86393**	0.83311**	0.54368	—	
Region H	0.87579**	0.74100**	0.89815**	0.86605**	0.29224**	0.66380**	0.77577**	—

* *p* < .05; **p < .01.

Neutrality and population expansion parameters for each gene are summarized in Table 5, Tables S7 and S8. For COI, we detected negative Tajima's *D* values for regions A (−2.1452), C (−2.6215), and D (−2.4064) with 99% statistical significance, indicating that the current haplotype diversity resulted from selection on certain genotypes. Tajima's *D* for regions B, E, F, and H was not statistically significant, indicating neutral evolution. The τ values that represent the estimated time of expansion were very low in regions A, B, C, and D (min = 0.0 in region B and max = 1.6 in region D), indicating sudden and recent population growth (Table 5). The τ values in regions E, F, and H were comparatively high (min = 9.2 in region E and max = 46.2 in region F), indicating that population growth was slower than that in regions A–D. The observed mismatch distribution was used to evaluate the demographic expansion history. The raggedness indexes for all regions except region E were not significant, suggesting that the expansion model could not be rejected, except in region E (Table 5). The analysis of region G, that is, the USA population, was not informative because the samples showed no haplotype variation.

**TABLE 5:** Neutrality test for COI

	Region (*N*_s_)								Mean ± *SD*
	A (36)	B (14)	C (24)	D (17)	E (23)	*F* (6)	G (3)	H (22)	
Tajima's *D*	−2.1452**	0.6124	−2.6215**	−2.4064**	1.6972	1.4576	—	1.0687	−0.2922 ± 1.8168
Tau (τ)	0.7	0.0	3.0	1.6	9.2	46.2	—	11.9	9.0728 ± 15.6562
SSD	0.0161	0.3451**	0.0124	0.0178**	0.1672	0.1568**	—	0.0854**	0.1001 ± 0.1192
Raggedness index	0.1052	0.3843	0.1952	0.1103	0.3297**	0.1867	—	0.1293	0.1801 ± 0.1253

Abbreviations: *N*_s_, number of samples; SSD, sum of squared deviation.

** *p* < .01.

### 3.3 Haplotype network

**FIGURE 1:**
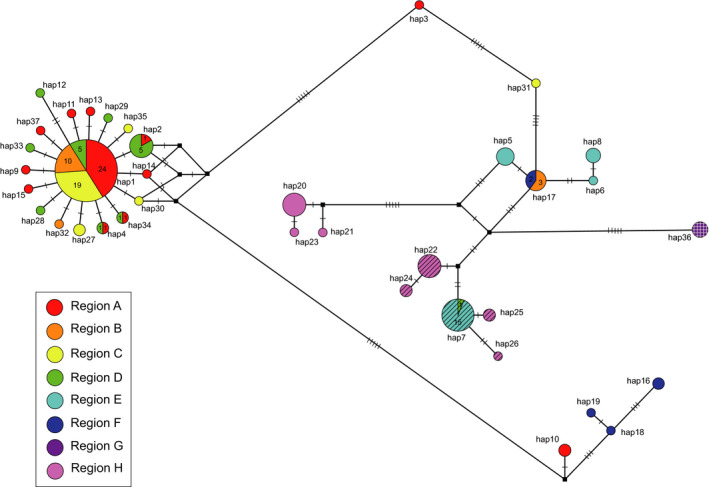
Haplotype network for COI. Circle size and line length are proportional to the haplotype frequency and the number of mutations, respectively. Geographic regions are color‐coded. Numbers on the pie charts indicate the number of individuals with the particular haplotypes in the region. The wing morph is represented by the following patterns: solid, L morph; diagonal stripe, S morph; crossed stripe, wing type unknown. Black square dots represent potentially missing haplotypes. The number of short vertical lines represents the number of mutational steps. Five short vertical lines indicate more than five mutational steps

In the haplotype network for COI, haplotype 1 was predominant in the Korean L morph samples, accounting for 40.0% of samples (58 individuals), including 41.4% of samples in region A, 32.8% in region C, 17.2% in region B, and 8.6% in region D (Figure 1). The USA samples belonged to haplotype 36 (Figure 1). Six haplotypes (haplotypes 1, 2, 4, 7, 17, and 34) were distributed in two or more regions and the other haplotypes were restricted to unique regions. Seventeen haplotypes were derived from haplotype 1, and 16 of these differed by a singleton mutation (Figure 1). Haplotype networks for COII and Cytb showed similar haplotype distribution patterns to that for COI (Figure S1). For all three genes, the Korean S morph haplotypes were more closely related to Japanese haplotypes than to the dominant L morph haplotypes in Korea (Figure 1; Figure S1).

### 3.4 Phylogenetic relationships and divergence time estimates

**FIGURE 2:**
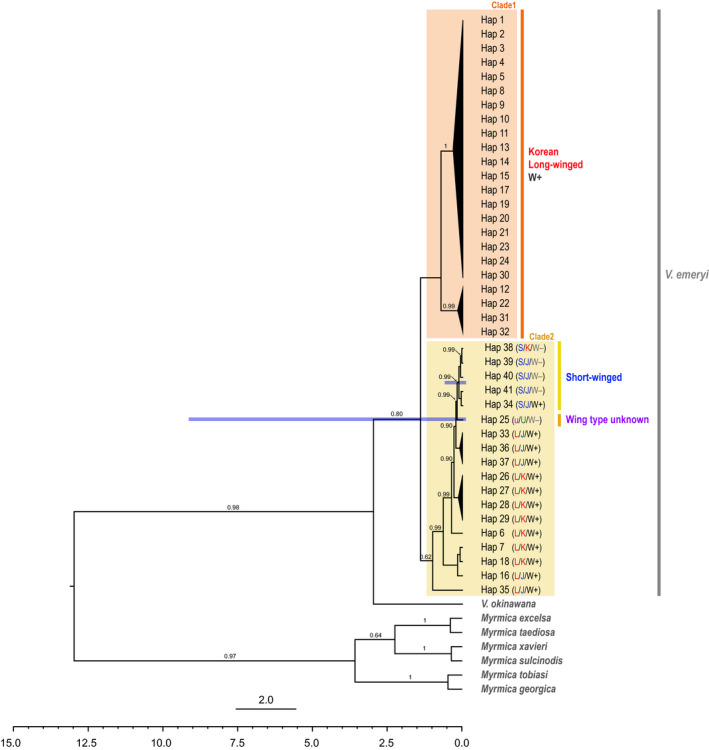
Estimation of divergence time between the S morph and the ancestral L morph haplotypes based on Bayesian inference using COI. Wing morph, country identity, and *Wolbachia* infection status are displayed: L = long‐winged, S = short‐winged, u = unknown, K = Korea, J = Japan, U = USA, W = *Wolbachia*. For clarity, all other node, error bars representing 95% HPD are removed, except those for the nodes of the two clades and the occurrence of the S morph haplotype

In the phylogenetic tree, we observed that the haplotypes were clearly divided into two clades, that is, clade 1 and clade 2, and the S morph was derived from the ancestral L morph (Figure 2). Clade 1 included only Korean L morph haplotypes, while clade 2 included haplotypes from Korea, Japan, and the USA, as well as both L and S morph haplotypes. The Korean and Japanese S morph haplotypes were monophyletic, implying that the wing transformation event took place only once in the history of the species. The USA haplotype (Hap 36) diverged earlier and was not monophyletic with the S morph haplotypes. Based on molecular dating, the two clades diverged approximately 2.7078 MYA (95% HPD: 0.0053–9.278 MYA), and the divergence of the S morph from the L morph occurred around 0.2 MYA (95% HPD: 0.0003–0.7164 MYA; Figure 2).

### 3.5 Wing morphology and *Wolbachia* infection

All of the L morph individuals proved to be infected with *Wolbachia*. However, *Wolbachia* infection was polymorphic in the S morph individuals. None of the Korean S morph populations (Hap 7) harbored *Wolbachia*, but the Japanese S morph populations collected from the mid‐northern part of Japan, that is, Ishikawa and Toyama (Hap 22), were completely infected whereas populations from Tokyo (Hap 25, 26) and Gifu (Hap 24) were free of *Wolbachia* (Figure 2). The wing development pattern correlated strongly with *Wolbachia* infection status in this ant species (*n* = 142, Pearson χ^2^ = 100.339, *df* = 1, *p* < .001). These results also suggest that *Wolbachia* is not involved in clonal reproduction in the ant species because clonal reproduction occurs in both wing morphs (Kobayashi et al., 2011).

### 3.6 COI clade and haplotype frequencies in the eight regions

**FIGURE 3:**
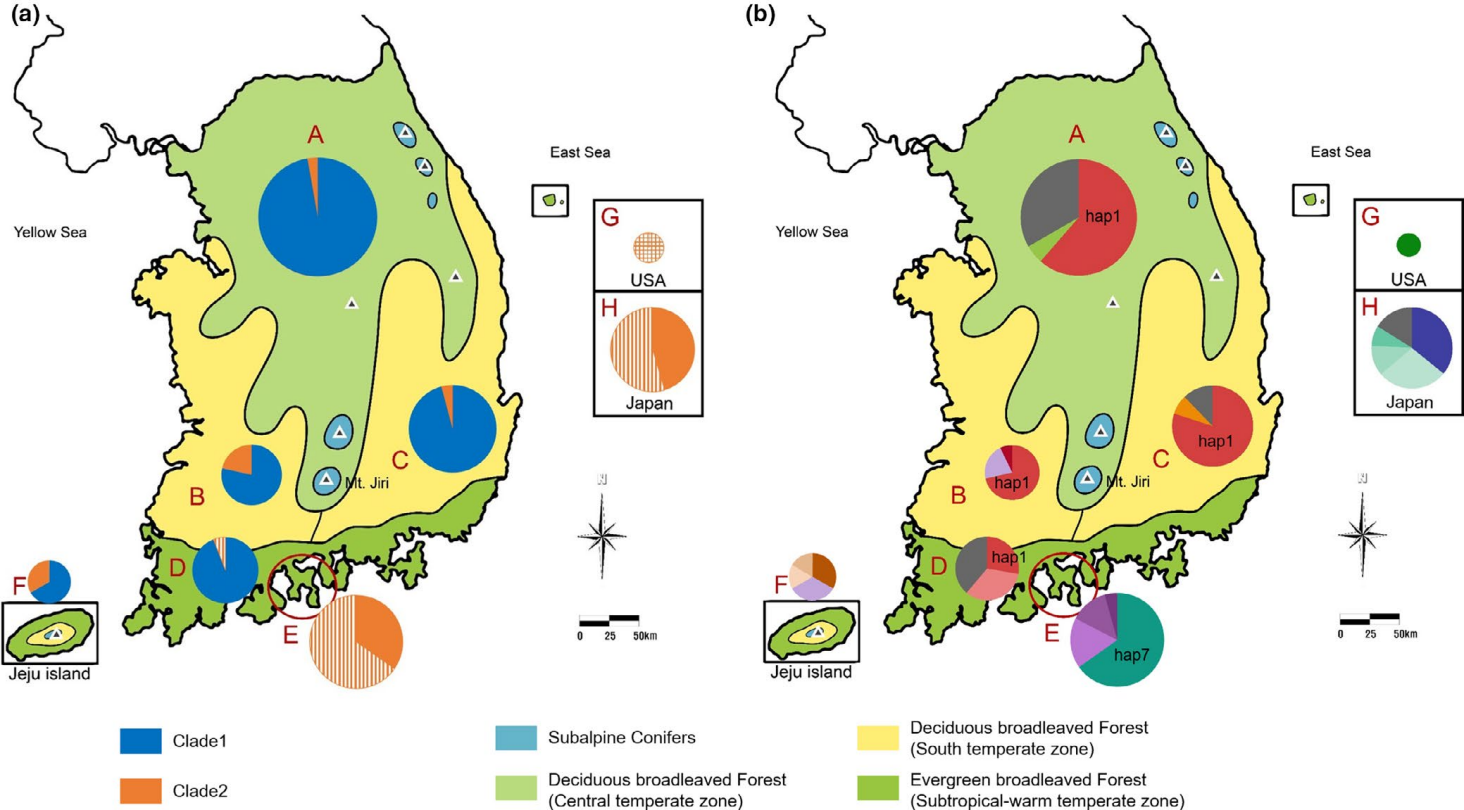
Vegetation map of South Korea, with pie charts (Yi, 2011). Pie size is proportional to the number of samples examined from each region. (a) Clade (color) and wing type (pattern) proportion are shown for each of six regions. Clade 1, blue; clade 2, orange; L morph, solid; S morph, stripe; wing type unknown, crossed stripe. (b) Haplotype proportion for each of six regions. The single haplotypes in each region are combined and shown in dark gray in the pies. The dominant haplotypes are presented on the pies

The COI haplotypes were divided into two clades in the Bayesian phylogenetic tree (Figure 2). The demarcated vegetation maps, with clade and haplotype composition data, are shown in Figure 3a. Regions A, C, and D showed similar ratios of clade 1 to clade 2. Clade 2 was slightly more highly represented in regions B and F than in regions A, C, and D. Even though region E belongs to the Korean peninsula, all haplotypes from the region formed a group with region H (Japan) and region G (USA) haplotypes in clade 2. Moreover, the ratio of the L morph to the S morph in region E was similar to that in region H (Japan).

Hap 1 was a dominant haplotype in regions A, B, and C. In region D, the frequency of hap 1 was lower than that in the other regions (Figure 3b). In Korean populations, region E (Yeosu‐si), in which the S morph can be found, and region *F* (Jeju), which is isolated from the mainland of the Korean peninsula, had haplotype compositions distant from those of regions A to D. The haplotype compositions and frequencies in region H (Japan) were different from those in Korea (Figure 3b).

## 4 DISCUSSION

The strong genetic isolation among regions (overall fixation index for COI: 0.781) indicates an extremely low dispersal rate after regional colonization, similar to the situation in Japan (Miyakawa & Mikheyev, 2015). In the Korean populations, other than the island (region F), pairwise *F*_ST_ values indicated more limited dispersal in the region E population than in the other populations; the S morph is found in this region, and its mating almost always occurs in the natal nest (Table 4; Ohkawara, Ishii, Fukushima, Yamauchi, & Heinze, 2002). The haplotype network and pairwise *F*_ST_ results indicate that populations in region E (the Korean S morph population) are closely related to populations in region H (Japan; Figure 1 and Table 4). In the COI phylogenetic tree, we detected a migration event(s) between region E and region H (Japan) after the S morph divaricated from the L morph (Figure 2). Our divergence estimation indicates that the emergence of S morph and loss of infection are evolutionarily very recent events (Figure 2). However, when interpreting these divergence data, caution is necessary because Hymenopteran insects show lineage‐specific variation with respect to mitochondrial evolution (Dowton, Cameron, Austin, & Whiting, 2009).

Populations in three regions (region A, C, and D) seem to be undergoing purifying selection, although mitochondrial DNA is a neutral marker (but see Morales, Pavlova, Joseph, & Sunnucks, 2015; Mossman, Jennifer, Navarro, & Rand, 2019; Table 5). Our results may be explained by the reproductive strategy and sib‐mating behavior. Selfish clonal reproduction forms strong maternal nuclear‐mitochondrial bonds in gynes, and sib‐mating behavior enhances paternal nuclear‐mitochondrial bondage in males, similar to linkage disequilibrium. Therefore, the signature of selection on a neutral marker may reflect selection for linked loci or nonrandomly associated genotypes.

Founder effect and relaxed selection may result in the loss of *Wolbachia* infection in some invasive ant species while exploring new habitats (Bouwma, Ahrens, DeHeer, & Shoemaker, 2006; Reuter, Pedersen, & Keller, 2005; Rey et al., 2013; Tsutsui, Kauppinen, Oyafuso, & Grosberg, 2003). In the endemic *V. emeryi* population in East Asia, the phylogenetic tree of the COI haplotypes shows that *Wolbachia* infection is evident in the ancestral L morph, but disappeared in the S morph (Figure 2). The speculation that the loss of *Wolbachia* of the S morph was caused by the colony founding process is less plausible because the S morph populations are endemic and the malformed wings of S morph queen restrict dispersal to a new habitats.

Łukasiewicz, Sanak, and Węgrzyn (2016) reported the correlation between malformation of wings and the absence of *Wolbachia* in apollo butterfly, *Parnassius apollo*. Their result is correspond to the phenomenon of the loss of *Wolbachia* in *V. emeryi* we investigated. In light of these results, it is reasonable to argue that the presence of *Wolbachia* be associated with wing development in these species. Although the mechanism of this connection remains to be elucidated, we suggest hypothesis from two perspectives, host or *Wolbachia* as a main driver of evolutionary outcome. In the former perspective, there might be a positive relationship between short wing formation and evolution of resistance to *Wolbachia* infection in this species. Epigenetic factors might be involved in wing formation in this ant based on intermittent L gyne production from the S morph colonies (Noh, 2014; Noh, Park, Choe, & Jeong, 2018; Okamoto, Kobayashi, Hasegawa, & Ohkawara, 2015). If that is the case, it is possible that the gene(s) responsible for the wing formation, and the gene(s) resistant to *Wolbachia* infection, exhibit epistatic interactions. Wing polymorphism is regulated by hormones mainly the juvenile hormone titer and certain genes (Zera, 2016). Such genes may encode antiogensin‐converting enzyme for bacterial immunity (Dani, Richards, Isaac, & Edwards, 2003). It will be meaningful to investigate such gene(s) to elucidate the prevalence of *Wolbachia* in insects from a mechanistic evolutionary perspective. Another speculation, in the latter perspective, is that *Wolbachia* might play a contributive role in the ontogenic stage of host wing development in these species, as suggested by Łukasiewicz et al. (2016). A further study that examines the effects of elimination of *Wolbachia* infection on these host species by antibiotic treatment will provide more insightful explanation for this uncommon association between host wing morphology and *Wolbachia* infection.

The *Wolbachia* bacterium is known for its manipulative effects on host reproduction (Fujii et al., 2004; Jeong & Suh, 2008; Stouthamer et al., 1999). The *Wolbachia*‐induced parthenogenesis is similar to queen developmental procedure in the ant *V. emeryi*. In the ant species, however, the clonal reproduction takes place in both *Wolbachia*‐infected L morph and *Wolbachia*‐free S morph. Therefore, *Wolbachia* may not contribute to clonal production system of the queen caste, as is the case in *W. auropunctata* (Rey et al., 2013).

In conclusion, all L morphs, the predominant ancestral form, were infected with *Wolbachia*, while the rare derived S morphs were free of *Wolbachia,* at least in Korean populations, and were partially infected in Japanese populations in parallel with the potential evolution of *Wolbachia* infection resistance. This is the significant report of an uncommon association between *Wolbachia* infection and host morphological characteristics.

## CONFLICT OF INTEREST

The authors have no conflict of interest.

## AUTHOR CONTRIBUTIONS

**Pureum Noh:** Data curation (lead); Formal analysis (lead); Investigation (lead); Methodology (lead); Software (lead); Visualization (lead); Writing‐original draft (lead). **Seung‐Yoon Oh:** Data curation (lead); Formal analysis (supporting); Investigation (lead); Methodology (supporting); Resources (lead); Software (supporting); Writing‐original draft (lead). **Soyeon Park:** Data curation (supporting); Investigation (supporting); Methodology (supporting); Resources (supporting); Software (supporting); Visualization (supporting); Writing‐original draft (supporting). **Taesung Kwon:** Methodology (supporting); Resources (supporting). **Yonghwan Kim:** Investigation (supporting); Methodology (supporting). **Jae Chun Choe:** Funding acquisition (supporting); Methodology (supporting); Project administration (supporting); Supervision (lead). **Gilsang Jeong:** Conceptualization (lead); Data curation (lead); Formal analysis (lead); Funding acquisition (lead); Investigation (lead); Methodology (lead); Project administration (lead); Resources (lead); Software (lead); Supervision (lead); Validation (lead); Writing‐original draft (lead); Writing‐review & editing (lead).

## Supporting information

Figure S1

Tables S1–S8

## Data Availability

Data are available at the Dryad Digital Repository, https://doi.org/10.5061/dryad.j6q573nb1. Sampling locations and GenBank accession numbers for the sequences of each sample are included in the Supporting information section (Table S1).

## References

[ece36582-bib-0001] Andersen, A. N. (1995). A classification of Australian ant communities, based on functional groups which parallel plant life‐forms in relation to stress and disturbance. Journal of Biogeography, 22(1), 15–29. 10.2307/2846070

[ece36582-bib-0002] Andersen, A. N. (1997). Functional groups and patterns of organization in North American ant communities: A comparison with Australia. Journal of Biogeography, 24, 433–460. 10.1111/j.1365-2699.1997.00137

[ece36582-bib-0003] Baldo, L. , Dunning Hotopp, J. C. , Jolley, K. A. , Bordenstein, S. R. , Biber, S. A. , Choudhury, R. R. , … Werren, J. H. (2006). Multilocus sequence typing system for the endosymbiont *Wolbachia pipientis* . Applied and Environmental Microbiology, 72(11), 7098–7110. 10.1128/aem.00731-06 16936055 PMC1636189

[ece36582-bib-0004] Bandelt, H.‐J. , Forster, P. , & Röhl, A. (1999). Median‐joining networks for inferring intraspecific phylogenies. Molecular Biology and Evolution, 16(1), 37–48. 10.1093/oxfordjournals.molbev.a026036 10331250

[ece36582-bib-0005] Bourtzis, K. , & Miller, T. A. (2008). Insect symbiosis. Boca Raton, FL: CRC Press.

[ece36582-bib-0006] Bouwma, A. M. , Ahrens, M. E. , DeHeer, C. J. , & Shoemaker, D. (2006). Distribution and prevalence of *Wolbachia* in introduced populations of the fire ant *Solenopsis invicta* . Insect Molecular Biology, 15(1), 89–93. 10.1111/j.1365-2583.2006.00614.x 16469072

[ece36582-bib-0007] Buschinger, A. , & Heinze, J. (1992). Polymorphism of female reproductives in ants. Paper presented at the First European Congress of Social Insects, Leuven (Belgium), 19–22 Aug 1991.

[ece36582-bib-0008] Correa, C. C. , & Ballard, J. W. O. (2016). *Wolbachia* associations with insects: Winning or losing against a master manipulator. Frontiers in Ecology and Evolution, 3, 153 10.3389/fevo.2015.00153

[ece36582-bib-0009] Dani, M. P. , Richards, E. H. , Isaac, R. E. , & Edwards, J. P. (2003). Antibacterial and proteolytic activity in venom from the endoparasitic wasp *Pimpla hypochondriaca* (Hymenoptera: Ichneumonidae). Journal of Insect Physiology, 49(10), 945–954. 10.1016/S0022-1910(03)00163-X 14511827

[ece36582-bib-0010] de Bekker, C. , Will, I. , Das, B. , & Adams, R. M. (2018). The ants (Hymenoptera: Formicidae) and their parasites: Effects of parasitic manipulations and host responses on ant behavioral ecology. Myrmecological News, 28, 1–24. 10.25849/myrmecol.news_028:001

[ece36582-bib-0011] Dowton, M. , Cameron, S. L. , Austin, A. D. , & Whiting, M. F. (2009). Phylogenetic approaches for the analysis of mitochondrial genome sequence data in the Hymenoptera–A lineage with both rapidly and slowly evolving mitochondrial genomes. Molecular Phylogenetics and Evolution, 52(2), 512–519. 10.1016/j.ympev.2009.04.001 19364540

[ece36582-bib-0012] Drummond, A. J. , & Rambaut, A. (2007). BEAST: Bayesian evolutionary analysis by sampling trees. BMC Evolutionary Biology, 7(1), 214 10.1002/9780471650126.dob0817 17996036 PMC2247476

[ece36582-bib-0013] Excoffier, L. , & Lischer, H. E. (2010). Arlequin suite ver 3.5: a new series of programs to perform population genetics analyses under Linux and Windows. Molecular Ecology Resources, 10(3), 564–567. 10.1111/j.1755-0998.2010.02847.x 21565059

[ece36582-bib-0014] Foucaud, J. , Estoup, A. , Loiseau, A. , Rey, O. , & Orivel, J. (2010). Thelytokous parthenogenesis, male clonality and genetic caste determination in the little fire ant: New evidence and insights from the lab. Heredity, 105(2), 205–212. 10.1038/hdy.2009.169 19935823

[ece36582-bib-0015] Foucaud, J. , Fournier, D. , Orivel, J. , Delabie, J. H. C. , Loiseau, A. , Le Breton, J. , … Estoup, A. (2007). Sex and clonality in the little fire ant. Molecular Biology and Evolution, 24(11), 2465–2473. 10.1093/molbev/msm180 17728279

[ece36582-bib-0016] Foucaud, J. , Jourdan, H. , Le Breton, J. , Loiseau, A. , Konghouleux, D. , & Estoup, A. (2006). Rare sexual reproduction events in the clonal reproduction system of introduced populations of the little fire ant. Evolution, 60(8), 1646–1657. 10.1554/06-099.1 17017065

[ece36582-bib-0017] Fournier, D. , Estoup, A. , Orivel, J. , Foucaud, J. , Jourdan, H. , Le Breton, J. , & Keller, L. (2005). Clonal reproduction by males and females in the little fire ant. Nature, 435(7046), 1230–1234. 10.1038/nature03705 15988525

[ece36582-bib-0018] Fu, Y. X. (1997). Statistical tests of neutrality of mutations against population growth, hitchhiking and background selection. Genetics, 147(2), 915–925. 10.1093/molbev/msl052 9335623 PMC1208208

[ece36582-bib-0019] Fujii, Y. , Kubo, T. , Ishikawa, H. , & Sasaki, T. (2004). Isolation and characterization of the bacteriophage WO from *Wolbachia*, an arthropod endosymbiont. Biochemical and Biophysical Research Communications, 317(4), 1183–1188. 10.1016/j.bbrc.2004.03.164 15094394

[ece36582-bib-0020] Heinze, J. , & Tsuji, K. (1995). Ant reproductive strategies. Researches on Population Ecology, 37(2), 135–149. 10.1007/bf02515814

[ece36582-bib-0021] Hilgenboecker, K. , Hammerstein, P. , Schlattmann, P. , Telschow, A. , & Werren, J. H. (2008). How many species are infected with *Wolbachia*?–a statistical analysis of current data. FEMS Microbiology Letters, 281(2), 215–220. 10.1111/j.1574-6968.2008.01110.x 18312577 PMC2327208

[ece36582-bib-0022] Ikeda, H. , Nishikawa, M. , & Sota, T. (2012). Loss of flight promotes beetle diversification. Nature Communications, 3(1), 1–8. 10.1038/ncomms1659 PMC3272566 22337126

[ece36582-bib-0023] Jansen, G. , & Savolainen, R. (2010). Molecular phylogeny of the ant tribe Myrmicini (Hymenoptera: Formicidae). Zoological Journal of the Linnean Society, 160(3), 482–495. 10.1111/j.1096-3642.2009.00604.x

[ece36582-bib-0024] Jeong, G. , & Stouthamer, R. (2004). Genetics of female functional virginity in the parthenogenesis‐*Wolbachia* infected parasitoid wasp *Telenomus nawai* (Hymenoptera: Scelionidae). Heredity, 94, 402 10.1038/sj.hdy.6800617 15523503

[ece36582-bib-0025] Jeong, G. , & Suh, E. (2008). *Wolbachia*‐induced reproductive anomalies and their future applications. Entomological Research, 38(1), 41–48. 10.1111/j.1748-5967.2008.00135.x

[ece36582-bib-0026] Kautz, S. , Rubin, B. E. , Russell, J. A. , & Moreau, C. S. (2013). Surveying the microbiome of ants: Comparing 454 pyrosequencing with traditional methods to uncover bacterial diversity. Applied and Environmental Microbiology, 79(2), 525–534. 10.1128/aem.03107-12 23124239 PMC3553759

[ece36582-bib-0027] Kinomura, K. , & Yamauchi, K. (1994). Frequent occurrence of gynandromorphs in the natural population of the ant *Vollenhovia emeryi* (Hymenoptera: Formicidae). Insectes Sociaux, 41(3), 273–278. 10.1007/bf01242298

[ece36582-bib-0028] Kjar, D. S. , & Suman, T. W. (2007). First records of invasion by the myrmicine Japanese ant *Vollenhovia emeryi* WM Wheeler (Hymenoptera: Formicidae) in the United States. Proceedings of the Entomological Society of Washington, 109(3), 596–604.

[ece36582-bib-0029] Kobayashi, K. , Hasegawa, E. , & Ohkawara, K. (2008). Clonal reproduction by males of the ant *Vollenhovia emeryi* (Wheeler). Entomological Science, 11(2), 167–172. 10.1111/j.1479-8298.2008.00272.x

[ece36582-bib-0030] Kobayashi, K. , Hasegawa, E. , & Ohkawara, K. (2011). No gene flow between wing forms and clonal reproduction by males in the long‐winged form of the ant *Vollenhovia emeryi* . Insectes Sociaux, 58(2), 163–168. 10.1007/s00040-010-0131-0

[ece36582-bib-0031] Kobayashi, K. , Tamura, K. , Okamoto, M. , Hasegawa, E. , & Ohkawara, K. (2012). Phylogenetic relationships among populations of *Vollenhovia* ants, with particular focus on the evolution of wing morphology. Annals of the Entomological Society of America, 105(3), 454–461. 10.1603/an11038

[ece36582-bib-0032] Kumar, S. , Nei, M. , Dudley, J. , & Tamura, K. (2008). MEGA: A biologist‐centric software for evolutionary analysis of DNA and protein sequences. Briefings in Bioinformatics, 9(4), 299–306. 10.1093/bib/bbn017 18417537 PMC2562624

[ece36582-bib-0033] Kumar, S. , Tamura, K. , & Nei, M. (1994). MEGA: Molecular evolutionary genetics analysis software for microcomputers. Computer Applications in the Biosciences: CABIOS, 10(2), 189–191. 10.1093/bioinformatics/10.2.189 8019868

[ece36582-bib-0034] Librado, P. , & Rozas, J. (2009). DnaSP v5: A software for comprehensive analysis of DNA polymorphism data. Bioinformatics, 25(11), 1451–1452. 10.1093/bioinformatics/btp187 19346325

[ece36582-bib-0035] Lin, X. , Yao, Y. , Wang, B. , Emlen, D. J. , & Lavine, L. C. (2016). Ecological trade‐offs between migration and reproduction are mediated by the nutrition‐sensitive insulin‐signaling pathway. International Journal of Biological Sciences, 12(5), 607 10.7150/ijbs.14802 27143957 PMC4852207

[ece36582-bib-0036] Lubertazzi, D. , & Tschinkel, W. (2003). Ant community change across a ground vegetation gradient in north Florida's longleaf pine flatwoods. Journal of Insect Science, 3(1), 21 10.1093/jis/3.1.21 15841237 PMC524660

[ece36582-bib-0037] Łukasiewicz, K. , Sanak, M. , & Węgrzyn, G. (2016). A lack of *Wolbachia*‐specific DNA in samples from apollo butterfly (*Parnassius apollo*, Lepidoptera: Papilionidae) individuals with deformed or reduced wings. Journal of Applied Genetics, 57(2), 271–274. 10.1007/s13353-015-0318-1 26423782 PMC4830866

[ece36582-bib-0038] Matsuura, K. (2010). Sexual and asexual reproduction in termites In Bignell D. E., Roisin Y., & Lo N. (Eds.), Biology of termites: A modern synthesis (pp. 255–277). Dordrecht, the Netherlands: Springer.

[ece36582-bib-0039] McCulloch, G. A. , Foster, B. J. , Dutoit, L. , Ingram, T. , Hay, E. , Veale, A. J. , … Waters, J. M. (2019). Ecological gradients drive insect wing loss and speciation: The role of the alpine treeline. Molecular Ecology, 28(13), 3141–3150. 10.1111/mec.15114 31038802

[ece36582-bib-0040] Miyakawa, M. O. , & Mikheyev, A. S. (2015). QTL mapping of sex determination loci supports an ancient pathway in ants and honey bees. PLoS Genetics, 11(11), e1005656 10.1371/journal.pgen.1005656 26544972 PMC4636138

[ece36582-bib-0041] Morales, H. E. , Pavlova, A. , Joseph, L. , & Sunnucks, P. (2015). Positive and purifying selection in mitochondrial genomes of a bird with mitonuclear discordance. Molecular Ecology, 24(11), 2820–2837. 10.1111/mec.13203 25876460

[ece36582-bib-0042] Mossman, J. A. , Jennifer, Y. G. , Navarro, F. , & Rand, D. M. (2019). Mitochondrial DNA fitness depends on nuclear genetic background in *Drosophila* . G3: Genes, Genomes, Genetics, 9(4), 1175–1188. 10.1534/g3.119.400067 30745378 PMC6469417

[ece36582-bib-0043] Noh, P. (2014). Population structure and microbiota of Vollenhovia emeryi Wheeler (Hymenoptera: Myrmicinae). MS thesis, Ewha Womans University.

[ece36582-bib-0044] Noh, P. , Park, S. Y. , Choe, J. C. , & Jeong, G. (2018). Genome size estimation of the two wing morphs of *Vollenhovia emeryi* (Hymenoptera: Myrmicinae). Korean Journal of Applied Entomology, 57(4), 317–322. 10.5656/KSAE.2018.10.0.044

[ece36582-bib-0045] Ohkawara, K. , Ishii, H. , Fukushima, Y. , Yamauchi, K. , & Heinze, J. (2002). Queen polymorphism and reproductive behaviour in myrmicine ant, *Vollenhovia emeryi* In Proceedings of XIV International Congress of IUSSI (p. 206). Sapporo, Japan: Hokkaido University Press.

[ece36582-bib-0046] Ohkawara, K. , Nakayama, M. , Satoh, A. , Trindl, A. , & Heinze, J. (2006). Clonal reproduction and genetic caste differences in a queen‐polymorphic ant, *Vollenhovia emeryi* . Biology Letters, 2(3), 359–363. 10.1098/rsbl.2006.0491 17148403 PMC1686177

[ece36582-bib-0047] Okamoto, M. , Kobayashi, K. , Hasegawa, E. , & Ohkawara, K. (2015). Sexual and asexual reproduction of queens in a myrmicine ant, *Vollenhovia emeryi* (Hymenoptera: Formicidae). Myrmecol News, 21, 13–17.

[ece36582-bib-0048] Pannebakker, B. A. , Pijnacker, L. P. , Zwaan, B. J. , & Beukeboom, L. W. (2004). Cytology of *Wolbachia*‐induced parthenogenesis in *Leptopilina clavipes* (Hymenoptera: Figitidae). Genome, 47(2), 299–303. 10.1139/g03-137 15060582

[ece36582-bib-0049] Pearcy, M. , Goodisman, M. A. , & Keller, L. (2011). Sib mating without inbreeding in the longhorn crazy ant. Proceedings of the Royal Society B: Biological Sciences, 278(1718), 2677–2681. 10.1098/rspb.2010.2562 PMC3136830 21288949

[ece36582-bib-0050] Peeters, C. (1991). Ergatoid queens and intercastes in ants: Two distinct adult forms which look morphologically intermediate between workers and winged queens. Insectes Sociaux, 38(1), 1–15. 10.1007/bf01242708

[ece36582-bib-0051] Peeters, C. (2012). Convergent evolution of wingless reproductives across all subfamilies of ants, and sporadic loss of winged queens (Hymenoptera: Formicidae). Myrmecological News, 16, 75–91.

[ece36582-bib-0052] Peeters, C. , & Ito, F. (2001). Colony dispersal and the evolution of queen morphology in social Hymenoptera. Annual Review of Entomology, 46(1), 601–630. 10.1146/annurev.ento.46.1.601 11112181

[ece36582-bib-0053] Pigneur, L. M. , Hedtke, S. M. , Etoundi, E. , & Van Doninck, K. (2012). Androgenesis: A review through the study of the selfish shellfish *Corbicula* spp. Heredity, 108(6), 581–591. 10.1038/hdy.2012.3 22473310 PMC3356815

[ece36582-bib-0054] Pontieri, L. , Schmidt, A. M. , Singh, R. , Pedersen, J. S. , & Linksvayer, T. A. (2017). Artificial selection on ant female caste ratio uncovers a link between female‐biased sex ratios and infection by *Wolbachia* endosymbionts. Journal of Evolutionary Biology, 30(2), 225–234. 10.1111/jeb.13012 27859964

[ece36582-bib-0055] Reuter, M. , Pedersen, J. S. , & Keller, L. (2005). Loss of *Wolbachia* infection during colonisation in the invasive Argentine ant *Linepithema humile* . Heredity, 94(3), 364–369. 10.1038/sj.hdy.6800601 15674380

[ece36582-bib-0056] Rey, O. , Estoup, A. , Facon, B. , Loiseau, A. , Aebi, A. , Duron, O. , … Foucaud, J. (2013). Distribution of endosymbiotic reproductive manipulators reflects invasion process and not reproductive system polymorphism in the little fire ant *Wasmannia auropunctata* . PLoS One, 8(3), e58467 10.1371/journal.pone.0058467 23505512 PMC3594316

[ece36582-bib-0057] Roff, D. A. (1986). The evolution of wing dimorphism in insects. Evolution, 40(5), 1009–1020. 10.2307/2408759 28556224

[ece36582-bib-0058] Russell, J. A. (2012). The ants (Hymenoptera: Formicidae) are unique and enigmatic hosts of prevalent *Wolbachia* (Alphaproteobacteria) symbionts. Myrmecological News, 16, 7–23.

[ece36582-bib-0059] Russell, J. A. , Funaro, C. F. , Giraldo, Y. M. , Goldman‐Huertas, B. , Suh, D. , Kronauer, D. J. C. , … Pierce, N. E. (2012). A veritable menagerie of heritable bacteria from ants, butterflies, and beyond: Broad molecular surveys and a systematic review. PLoS One, 7(12), e51027 10.1371/journal.pone.0051027 23284655 PMC3527441

[ece36582-bib-0078] Simon, C. , Frati, F. , Beckenbach, A. , Crespi, B. , Liu, H. , & Flook, P. (1994). Evolution, weighting, and phylogenetic utility of mitochondrial gene sequences and a compilation of conserved polymerase chain reaction primers.. Annals of the entomological Society of America, 87(6), 651–701. 10.1093/aesa/87.6.651

[ece36582-bib-0060] Singh, R. , & Linksvayer, T. A. (2020). *Wolbachia*‐infected ant colonies have increased reproductive investment and an accelerated life cycle. Journal of Experimental Biology, 223(Pt 9), jeb.220079 10.1242/jeb.220079 32253286

[ece36582-bib-0061] Slatkin, M. (1987). Gene flow and the geographic structure of natural populations. Science (Washington), 236(4803), 787–792. 10.1126/science.3576198 3576198

[ece36582-bib-0062] Slatkin, M. , & Barton, N. H. (1989). A comparison of three indirect methods for estimating average levels of gene flow. Evolution, 43(7), 1349–1368. 10.2307/2409452 28564250

[ece36582-bib-0063] Stouthamer, R. , Breeuwer, J. A. , & Hurst, G. D. (1999). *Wolbachia pipientis*: Microbial manipulator of arthropod reproduction. Annual Reviews in Microbiology, 53(1), 71–102. 10.1146/annurev.micro.53.1.71 10547686

[ece36582-bib-0064] Tajima, F. (1989). Statistical method for testing the neutral mutation hypothesis by DNA polymorphism. Genetics, 123(3), 585–595. 2513255 10.1093/genetics/123.3.585 PMC1203831

[ece36582-bib-0065] Thompson, J. D. , Higgins, D. G. , & Gibson, T. J. (1994). CLUSTAL W: Improving the sensitivity of progressive multiple sequence alignment through sequence weighting, position‐specific gap penalties and weight matrix choice. Nucleic Acids Research, 22(22), 4673–4680. 10.1093/nar/22.22.4673 7984417 PMC308517

[ece36582-bib-0066] Tinaut, A. , & Heinze, J. (1992). Wing reduction in ant queens from arid habitats. Naturwissenschaften, 79(2), 84–85. 10.1007/bf01131809

[ece36582-bib-0067] Tsutsui, N. D. , Kauppinen, S. N. , Oyafuso, A. F. , & Grosberg, R. K. (2003). The distribution and evolutionary history of *Wolbachia* infection in native and introduced populations of the invasive argentine ant (*Linepithema humile*). Molecular Ecology, 12(11), 3057–3068. 14629385 10.1046/j.1365-294x.2003.01979.x

[ece36582-bib-0068] Villet, M. H. (1991). Colony foundation in *Plectroctena mandibularis* F. Smith, and the evolution of ergatoid queens in *Plectroctena* (Hymenoptera: Formicidae). Journal of Natural History, 25(4), 979–983. 10.1080/00222939100770641

[ece36582-bib-0069] Wenseleers, T. , Sundström, L. , & Billen, J. (2002). Deleterious *Wolbachia* in the ant *Formica truncorum* . Proceedings of the Royal Society of London. Series B: Biological Sciences, 269(1491), 623–629. 10.1098/rspb.2001.1927 11916479 PMC1690935

[ece36582-bib-0070] Werren, J. H. (1997). Biology of *Wolbachia* . Annual Review of Entomology, 42(1), 587–609. 10.1146/annurev.ento.42.1.587 15012323

[ece36582-bib-0071] Werren, J. H. , & Windsor, D. M. (2000). Wolbachia infection frequencies in insects: evidence of a global equilibrium? Proceedings of the Royal Society of London B: Biological Sciences, 267(1450), 1277–1285. 10.1098/rspb.2000.1139 PMC1690679 10972121

[ece36582-bib-0072] Wetterer, J. K. , Guenard, B. , & Booher, D. B. (2015). Geographic spread of *Vollenhovia emeryi* (Hymenoptera: Formicidae). Asian Myrmecology, 7(1), 105–112.

[ece36582-bib-0073] Wright, J. B. , & Kubik, E. (2011). A New Locality Record for *Vollenhovia emeryi* WM Wheeler (Hymenoptera: Formicidae) in Maryland, USA. Entomological News, 122(2), 170–172. 10.3157/021.122.0210

[ece36582-bib-0074] Yi, S. (2011). Holocene vegetation responses to East Asian monsoonal changes in South Korea In Blanco J., & Kheradmand H. (Eds.), Climate change–Geophysical foundations and ecological effects (pp. 157–178). Rijeka, Croatia: InTech.

[ece36582-bib-0075] Zera, A. J. (2016). Juvenile Hormone and the endocrine regulation of wing polymorphism in insects: New insights from circadian and functional‐genomic studies in *Gryllus* crickets. Physiological Entomology, 41(4), 313–326. 10.1111/phen.12166

[ece36582-bib-0076] Zientz, E. , Feldhaar, H. , Stoll, S. , & Gross, R. (2005). Insights into the microbial world associated with ants. Archives of Microbiology, 184(4), 199–206. 10.1007/s00203-005-0041-0 16205909

[ece36582-bib-0077] Zug, R. , & Hammerstein, P. (2015). Bad guys turned nice? A critical assessment of *Wolbachia* mutualisms in arthropod hosts. Biological Reviews, 90(1), 89–111. 10.1111/brv.12098 24618033

