# Supplementary figures and images for: Association between host wing morphology polymorphism and Wolbachia infection in Vollenhovia emeryi (Hymenoptera: Myrmicinae)
Source: Ecol Evol. 2020 Jul 28;10(16):8827–37. doi: 10.1002/ece3.6582 (PMC7452775; doi:10.1002/ece3.6582)

(a)

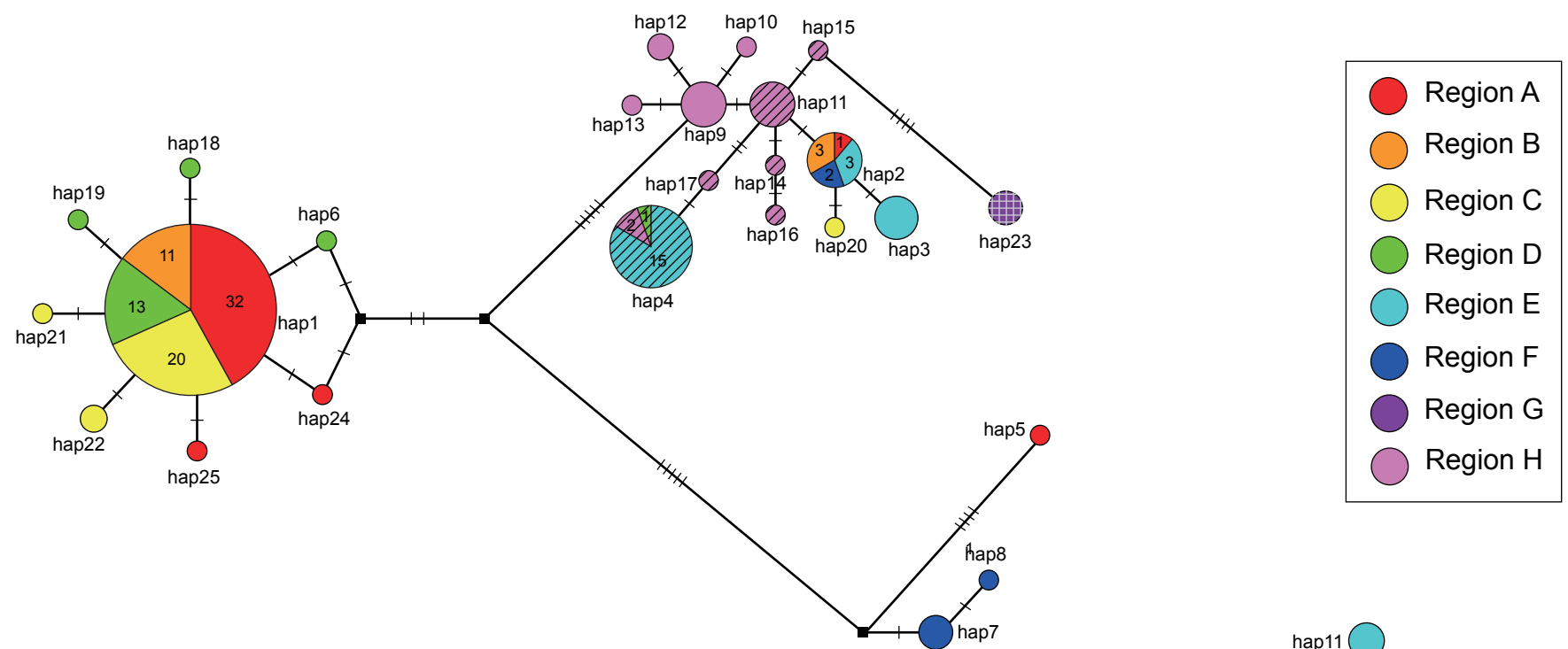

(b)

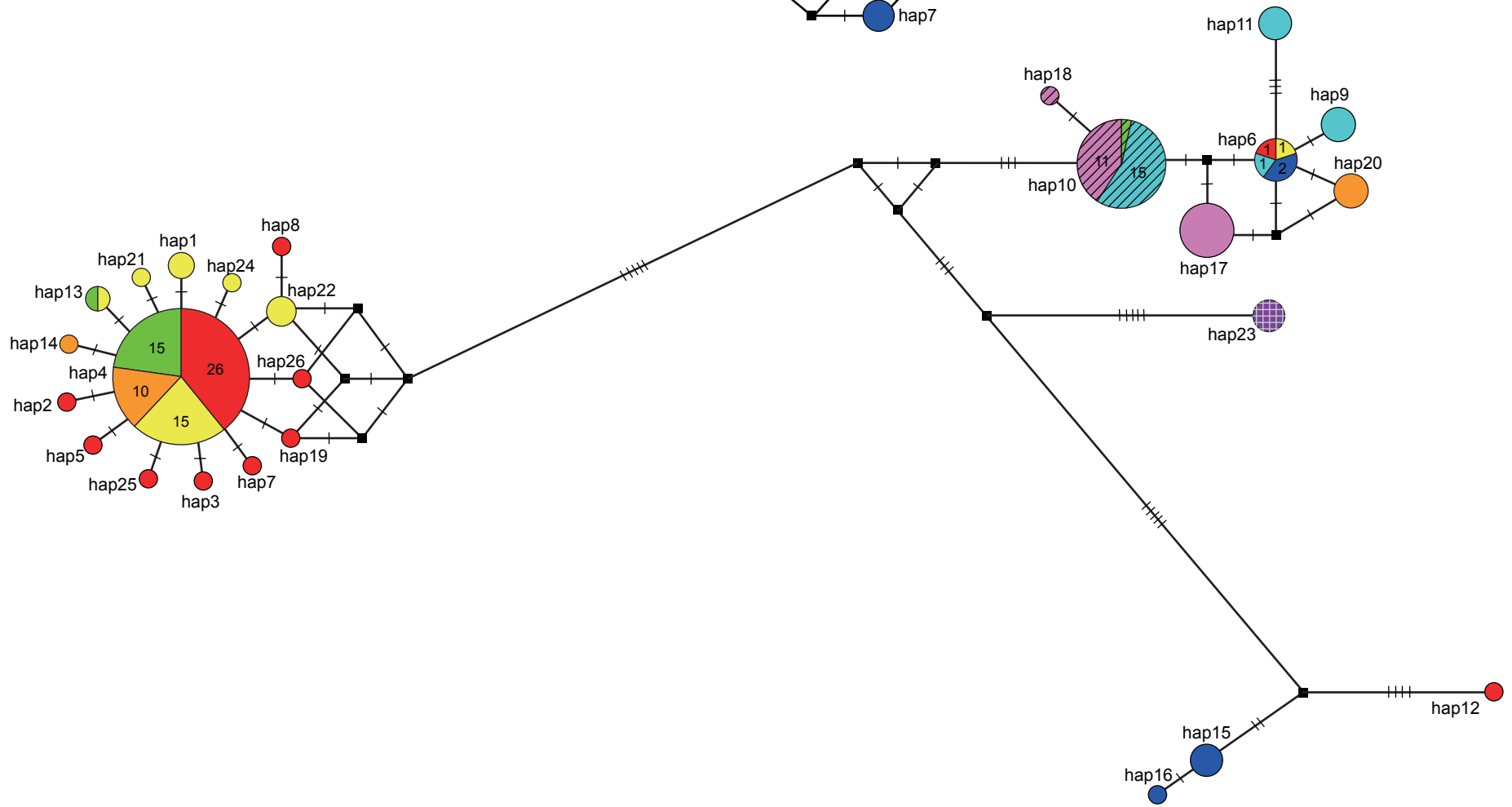

Supplement: Supplementary file 1 — Figure S1 [file ECE3-10-8827-s001.pdf]
