# Supplementary material for: Association between host wing morphology polymorphism and Wolbachia infection in Vollenhovia emeryi (Hymenoptera: Myrmicinae)
Source: Ecol Evol. 2020 Jul 28;10(16):8827–37. doi: 10.1002/ece3.6582 (PMC7452775; doi:10.1002/ece3.6582)
Supplement: Supplementary file 2 — Tables S1–S8 [file ECE3-10-8827-s002.docx]

**Supporting Information for:**

**Population structure reveals an association between host wing morphology polymorphism and *Wolbachia* infection in *Vollenhovia emeryi* (Hymenoptera: Myrmicinae)**

PUREUM NOH^1,6,a^, SEUNG-YOON OH^2^, SOYEON PARK^3,6^ TAESUNG KWON^4^, YONGHWAN KIM^5^, JAE CHUN CHOE^1^, and GILSANG JEONG^1,6,*^

^1^Division of EcoScience, Ewha Womans University, Seoul 03766, Korea;

^2^School of Biological Sciences, Seoul National University, Seoul 08826, Korea;

^3^Interdisciplinary Program of EcoCreative, The Graduate School, Ewha Womans University, Seoul 03766, Korea;

^4^Division of Forest Ecology, Korea Forest Research Institute, Seoul 02455, Korea;

^5^Department of Physics, Konkuk University, Seoul 143701, Korea;

^6^National Institute of Ecology, Seochun-gun 33657, Korea

*Correspondence: Gilsang Jeong, National Institute of Ecology, Seochun-gun 33657, Korea. Fax: +82 41 950 5953; E-mail: gilsangj@nie.re.kr

^a^Current affiliation: K-herb Research Center, Korea Institute of Oriental Medicine, Daejeon 34054, Korea

| Table of Contents | |
| --- | --- |
| Table S1 ………………………………. | Page 1–2 |
| Table S2 ………………………………. | Page 3–6 |
| Table S3 ………………………………. | Page 7 |
| Table S4 ………………………………. | Page 8 |
| Table S5 ………………………………. | Page 9 |
| Table S6 ………………………………. | Page 9 |
| Table S7 ………………………………. | Page 10 |
| Table S8 ………………………………. | Page 10 |
| Figure S1 ………………………………. | Page 11 |

**Table S1**. Information for *V. emeryi* samples

| Country | Region | *Vc* | Location | Latitude | *Cd* | *Nc* (COI hap) | GenBank accession number | | |
| --- | --- | --- | --- | --- | --- | --- | --- | --- | --- |
|  |  |  |  |  |  |  | COI | COII | Cytb |
| South  Korea | Region A | AS | Ansan-si, Gyeonggi-do | 37°19’N 126°52’E | Jun-11 | 1 (hap1) | KF815789 | MG206091 | MG254906 |
|  |  | BA | Seoul | 37°39’N 127°06’E | Jun-12 | 1 (hap1) | KF815821 | MG206092 | MG254907 |
|  |  | BG | Seongnam-si, Gyeonggi-do | 37°21’N 127°07’E | Jun-11 | 2 (hap1) | KF815781-2 | MG206093-4 | MG254908-9 |
|  |  | CA | Cheonan-si, Chungcheongnam-do | 36°48’N 127°05’E | Jun-11 | 1 (hap3) | KF815791 | MG206097 | MG254912 |
|  |  | CG | Uiwang-si, Gyeonggi-do | 37°24’N 127°02’E | Jun-11 | 1 (hap4) | KF815780 | MG206098 | MG254913 |
|  |  | CS | Chuncheon-si, Gangwon-do | 37°54’N 127°49’E | Jul-11 | 1 (hap2) | KF815748 | MG206099 | MG254914 |
|  |  | DB | Seoul | 37°40’N 127°02’E | Sep-13 | 1 (hap1) | KF815856 | MG206100 | MG254915 |
|  |  | DY | Muju-gun, Jeollabuk-do | 35°53’N 127°45’E | Jul-11 | 2 (hap1, 9) | KF815758-9 | MG206125-6 | MG254940-1 |
|  |  | GC | Gwacheon-si, Gyeonggi-do | 37°25’N 127°01’E | Jun-11 | 1 (hap10) | KF815779 | MG206127 | MG254942 |
|  |  | GKM | Suwon-si, Gyeonggi-do | 37°19’N 127°00’E | Jul-11 | 5 (hap1, 10, 11) | KF815797-801 | MG206134-8 | MG254949-53 |
|  |  | GP | Gapyeong-gun, Gyeonggi-do | 37°51’N 127°32’E | Jun-11 | 1 (hap1) | KF815747 | MG206139 | MG254954 |
|  |  | HY | Gapyeong-gun, Gyeonggi-do | 37°41’N 127°27’E | Sep-12 | 1 (hap13) | KF815802 | MG206143 | MG254958 |
|  |  | IC | Incheon | 37°30’N 126°41’E | Jun-11 | 1 (hap14) | KF815785 | MG206144 | MG254959 |
|  |  | IJ | Inje-gun, Gangwon-do | 38°04’N 128°10’E | Jun-11 | 1 (hap1) | KF815743 | MG206145 | MG254960 |
|  |  | JG | Gwangju-si, Gyeonggi-do | 37°20’N 127°22’E | Jul-11 | 1 (hap15) | KF815786 | MG206146 | MG254961 |
|  |  | JR | Sancheong-gun, Gyeongsangnam-do | 35°22’N 127°53’E | Jul-11 | 2 (hap1) | KF815767-8 | MG206175-6 | MG254990-1 |
|  |  | OS | Osan-si, Gyeonggi-do | 37°08’N 127°05’E | Jun-11 | 1 (hap1) | KF815790 | MG206188 | MG255003 |
|  |  | PC | Pocheon-si, Gyeonggi-do | 37°54’N 127°10’E | Jun-11 | 1 (hap1) | KF815744 | MG206189 | MG255004 |
|  |  | SDM | Gapyeong-gun, Gyeonggi-do | 37°54’N 127°32’E | Jul-11 | 1 (hap1) | KF815745 | MG206202 | MG255017 |
|  |  | SR | Boeun-gun, Chungcheongbuk-do | 36°31’N 127°48’E | Jul-11 | 1 (hap34) | KF815784 | MG206206 | MG255021 |
|  |  | SRV | Gunpo-si, Gyeonggi-do | 37°21’N 126°54’E | Jun-11 | 1 (hap1) | KF815796 | MG206207 | MG255022 |
|  |  | TJH | Seongnam-si, Gyeonggi-do | 37°21’N 127°08’E | Jul-11 | 1 (hap1) | KF815783 | MG206211 | MG255026 |
|  |  | WJ | Wonju-si, Gangwon-do | 37°20’N 127°55’E | Jun-11 | 1 (hap37) | KF815750 | MG206221 | MG255036 |
|  |  | YG | Yanggu-gun, Gangwon-do | 38°06’N 127°59’E | Jun-11 | 1 (hap1) | KF815749 | MG206227 | MG255042 |
|  |  | YIM | Gapyeong-gun, Gyeonggi-do | 37°54’N 127°33’E | Jul-11 | 1 (hap1) | KF815746 | MG206228 | MG255043 |
|  |  | YJ | Yeoju-gun, Gyeonggi-do | 37°17’N 127°38’E | Jun-11 | 2 (hap1) | KF815787-8 | MG206229-30 | MG255044-5 |
|  |  | YK | Sejong-si | 36°37’N 127°16’E | Jun-11 | 1 (hap1) | KF815792 | MG206231 | MG255046 |
|  |  | YW | Yeongwol-gun, Gangwon-do | 37°10’N 128°27’E | Jun-11 | 1 (hap1) | KF815751 | MG206234 | MG255049 |
|  | Region B | HS | Hongseong-gun, Chungcheongnam-do | 36°34’N 126°38’E | Jun-11 | 1 (hap1) | KF815794 | MG206142 | MG254957 |
|  |  | KJ | Gimje-si, Jeollanam-do | 35°50’N 126°49’E | Jul-11 | 1 (hap1) | KF815761 | MG206182 | MG254997 |
|  |  | PD | Ansan-si, Gyeonggi-do | 37°06’N 126°23’E | May-13 | 4 (hap1) | KF815814-8 | MG206190-3 | MG255005-8 |
|  |  | SC | Sunchang-gun, Jeollabuk-do | 35°23’N 127°07’E | Jul-11 | 6 (hap1, 17) | KF815825-30 | MG206196-201 | MG255011-6 |
|  |  | SE | Seocheon-gun, Chungcheongnam-do | 36°05’N 126°41’E | Jun-11 | 2 (hap1, 32) | KF815777-8 | MG206203-4 | MG255018-9 |
|  | Region C | AD | Andong-si, Gyeongsangbuk-do | 36°34’N 128°42’E | Aug-13 | 1 (hap1) | KF815840 | MG206090 | MG254905 |
|  |  | GJ | Ulju-gun, Ulsan | 35°37’N 129°00’E | Jul-11 | 2 (hap1) | KF815771-2 | MG206132-3 | MG254947-8 |
|  |  | JW | Cheongsong-gun, Gyeongsangbuk-do | 36°24’N 129°09’E | Jul-13 | 2 (hap1, 27) | KF815845-6 | MG206177-8 | MG254992-3 |
|  |  | KS | Gyeongsan-si, Gyeongsangbuk-do | 35°48’N 128°45’E | Jul-11 | 1 (hap30) | KF815776 | MG206183 | MG254998 |
|  |  | MG | Mungyeong-si, Gyeongsangbuk-do | 36°39’N 128°07’E | Aug-13 | 1 (hap1) | KF815847 | MG206184 | MG254999 |
|  |  | NHC | Hapcheon-gun, Gyeongsangnam-do | 35°34’N 128°08’E | Jul-11 | 1 (hap31) | KF815760 | MG206185 | MG255000 |
|  |  | PH | Pohang-si, Gyeongsangbuk-do | 36°03’N 129°18’E | Jun-11 | 1 (hap1) | KF815773 | MG206194 | MG255009 |
|  |  | SB | Ulju-gun, Ulsan | 35°32’N 129°03’E | Jul-11 | 1 (hap1) | KF815769 | MG206195 | MG255010 |
|  |  | SS | Gumi-si, Gyeongsangbuk-do | 36°15’N 128°16’E | Jun-11 | 2 (hap1, 35) | KF815765-6 | MG206208-9 | MG255023-4 |
|  |  | TH | Gyeongju-si, Gyeongsangbuk-do | 35°45’N 129°22’E | Jul-11 | 1 (hap1) | KF815770 | MG206210 | MG255025 |
|  |  | UL | Ulleung-gun, Gyeongsangbuk-do | 37°30’N 130°51’E | Jul-12 | 5 (hap1, 27) | KF815803-7 | MG206213-7 | MG255028-32 |
|  |  | YC | Yeongcheon-si, Gyeongsangbuk-do | 35°57’N 128°58’E | Jun-11 | 1 (hap1) | KF815774 | MG206222 | MG255037 |
|  |  |  |  | 36°07’N 128°57’E | Jul-13 | 1 (hap1) | KF815775 | MG206223 | MG255038 |
|  |  | YDK | Yeongdeok-gun, Gyeongsangbuk-do | 36°19’N 129°22’E | Jul-13 | 2 (hap1) | KF815848-9 | MG206225-6 | MG255040-1 |
|  |  | YNJ | Yeongju-si, Gyeongsangbuk-do | 36°48’N 128°36’E | Aug-13 | 2 (hap1) | KF815850-1 | MG206232-3 | MG255047-8 |
|  | Region D | BR | Gangjin-gun, Jeollanam-do | 34°35’N 126°44’E | Jul-11 | 2 (hap2) | KF815752-3 | MG206095-6 | MG254910-1 |
|  |  | DH | Haenam-gun, Jeollanam-do | 34°28’N 126°37’E | Aug-11 | 1 (hap1) | KF815757 | MG206101 | MG254916 |
|  |  | GD | Busan | 35°03’N 128°49’E | Jul-13 | 2 (hap1, 2) | KF815841-2 | MG206128-9 | MG254943-4 |
|  |  | GG | Gangjin-gun, Jeollanam-do | 34°39’N 126°45’E | Jul-11 | 2 (hap1) | KF815754-5 | MG206130-1 | MG254945-6 |
|  |  | GR | Geoje-si, Gyeongsangnam-do | 34°52’N 128°36’E | Jul-13 | 2 (hap1, 12) | KF815843-4 | MG206140-1 | MG254955-6 |
|  |  | KH | Gimhae-si, Gyeongsangnam-do | 35°14’N 128°55’E | Jul-11 | 1 (hap28) | KF815818 | MG206179 | MG254994 |
|  |  |  |  | 35°15’N 128°52’E | May-13 | 2 (hap4, 29) | KF815819-20 | MG206180-1 | MG254995-6 |
|  |  | OD | Yeosu-si, Jeollanam-do | 34°44’N 127°45’E | Aug-11 | 2 (hap2) | KF815812-3 | MG206186-7 | MG255001-2 |
|  |  | SJ | Gangjin-gun, Jeollanam-do | 34°41’N 126°42’E | Aug-11 | 1 (hap33) | KF815756 | MG206205 | MG255020 |
|  |  | TY | Tongyeong-si, Gyeongsangnam-do | 34°53’N 128°24’E | **Jul-13** | **1 (hap7)** | **KF815885** | **MG206212** | **MG255027** |
|  |  | YD | Mokpo-si, Jeollanam-do | 34°47’N 126°22’E | Jul-11 | 2 (hap34) | KF815764 | MG206224 | MG255039 |
|  | Region E | DS | Yeosu-si, Jeollanam-do | 34°39’N 127°45’E | Aug-12 | 8 (hap5, 6, 8) | KF815831-7 | MG206121-4,  MG206102-3,  MG206105 | MG254936-9,  MG254917-8,  MG254920 |
|  |  |  |  | 34°43’N 127°44’E | **Jul-11** | **1 (hap7)** | **KF815870** | **MG206120** | **MG254935** |
|  |  |  |  |  | Aug-12 | 1 (hap8) | KF815838 | MG206109 | MG254924 |
|  |  |  |  |  | **Aug-12** | **5 (hap7)** | **KF815871-5** | **MG206104**  **MG206106-8**  **MG206110** | **MG254919,**  **MG254921-3,**  **MG254925** |
|  |  |  |  |  | **Sep-12** | **4 (hap7)** | **KF815876-9** | **MG206111-4** | **MG254926-9** |
|  |  |  |  |  | **Apr-13** | **5 (hap7)** | **KF815880-4** | **MG206115-9** | **MG254930-4** |
|  | Region F | JJ | Jeju-si, Jeju-do | 33°28’N 126°29’E | Aug-11 | 2 (hap16) | KF815810-1 | MG206147-8 | MG254962-3 |
|  |  |  |  | 33°24’N 126°24’E | Sep-13 | 1 (hap18) | KF815854 | MG206151 | MG254966 |
|  |  |  | Seogwipo-si, Jeju-do | 33°18’N 126°34’E | Sep-13 | 1 (hap17) | KF815852 | MG206149 | MG254964 |
|  |  |  |  | 33°18’N 126°35’E | Sep-13 | 1 (hap17) | KF815853 | MG206150 | MG254965 |
|  |  |  |  | 33°19’N 126°36’E | Sep-13 | 1 (hap19) | KF815855 | MG206152 | MG254967 |
| USA | Region G | US | Washington DC, United States | 38°54’N 77°03 W | Jul-2013 | 3 (hap36) | KF815822-4 | MG206218-20 | MG255033-5 |
| Japan | Region H | JP | Shiga prefecture | 34°58’N 135°52’E | Aug-13 | 1 (hap20) | KF815857 | MG206153 | MG254968 |
|  |  |  | Kyoto prefecture | 35°02’N 135°47’E | Apr-13 | 1 (hap20) | KF815858 | MG206157 | MG254972 |
|  |  |  | Kyoto prefecture | 35°02’N 135°47’E | Aug-13 | 1 (hap23) | KF815861 | MG206159 | MG254974 |
|  |  |  | Kyoto prefecture | 35°01’N 135°47’E | **Oct-12** | **1 (hap22)** | **KF815859** | **MG206158** | **MG254973** |
|  |  |  | Kyoto prefecture | 35°03’N 135°43’E | Oct-13 | 4 (hap20, 21) | KF815867-9 | MG206154-6 | MG254969-71 |
|  |  |  | Hyogo prefecture | 34°44’N 135°15’E | Oct-13 | 4 (hap20) | KF815862-5 | MG206160-3 | MG254975-8 |
|  |  |  | Ishikawa prefecture | 36°34’N 136°33’E | **2013** | **3 (hap22)** | **KF815886-8** | **MG206164**  **MG206167-8** | **MG254979,**  **MG254982-3** |
|  |  | JPS | Tokyo | 35°45’N 139°18’E | **2013** | **3 (hap25, 26)** | **KF815889-91** | **MG206169-71** | **MG254984-6** |
|  |  |  | Toyama prefecture | 36°44’N 137°01’E | **2009** | **3 (hap22)** | **KF815892-4** | **MG206172-4** | **MG254987-9** |
|  |  |  | Gifu prefecture | NO DATA | **2010** | **3 (hap24)** | **KF815895,**  **KF815897** | **MG206165-6** | **MG254980-1** |
|  |  | VO | Okinawa prefecture | 28°58’N 128°01’E | Sep-2012 |  | KF815898 | - | - |
| *Vc*, Voucher Code; *Cd*, Collection date; *Nc*, Number of colonies. Bold letters denote S morph colonies. | | | | | | | | | |

**Table S2.** Variants in the mitochondrial COI, COII, and Cytb haplotypes of *V. emeryi*

| Gene | Haplotype ID | Sequence |
| --- | --- | --- |
| COI | Hap 1 | AAACATAGCGAATTACATCTTGTATTCTAGCCCTTTCGTGACGGCTCTATCGCCTAGCAGGCCTTATAAGGCACTTTCCCCCACATTCTATGAATATATTCGCG |
|  | Hap 2 | AAACATAGCGAATTACATCTTGTATTCTAGCCCTTTCGTGACGGCTCTATCGCCTAGCAGGCCTTATAAGGCACTTTCCCCCACATTCTGTGAATATATTCGCG |
|  | Hap 3 | AAACATAGCGAATTACGTCTTGTGTTCTGGCCCTTTCATAATGGCTCCGTTACCTAGTAAGTCCCACAGGGCATCTCCCCCCGCACTTCGCAGACGCACTCGTT |
|  | Hap 4 | AAACATAGCGAATTACATCTTGTATTCTAGCCCTTTCGTGACGGCTCTATCGCCTAGCAGGCCTTATAAGGCACTTTCCCCCACATTCTATGAATATATTCGCA |
|  | Hap 5 | AAACACGATCAACCACGTCCCACGTCTCGACCCTTCCATAATGGCTTCGTTACCTAGTAAGTCCCACAGGGCATCTCCCCCCGCACTTCGCAGACGCACTCGTT |
|  | Hap 6 | AAACACGATCAACCACGTCCCACGTCTCGACCCTTCCATAATAGCTCCGTTACCTAGTAAGTCCCACAGGGCATCTCCCCCCGCGCTTCGCAGACGCACTCGTT |
|  | Hap 7 | AAACACGATCAGCCACGTCCCACATCTCGACTCCTCCATAATGGCTCCGTTACCTAGCAAGTCCCACAGGGCATCTCCCCCCACACTTCGCAGACGCACCCGTT |
|  | Hap 8 | AAACACGATCAACCACGTCCCACGTCTCGACCCTTCCATAATAGCTCCGTTATCTAGTAAGTCCCACAGGGCATCTCCCCCCGCGCTTCGCAGACGCACTCGTT |
|  | Hap 9 | AAACATAGCGAATTACATCTTGTATTCTAGCCCTTTCGTGACGACTCTATCGCCTAGCAGGCCTTATAAGGCACTTTCCCCCACATTCTATGAATATATTCGCG |
|  | Hap 10 | AAATGTGACGGACTGCGTTCTGTATCTTAACCCTCCCATAACGGTTCCATCGCTCTGCCAGTTCTATAAAATACCTCCTCCTACATTCCATAGACATATTCGTG |
|  | Hap 11 | AAACATAGCGAATTACATCTTGTATTCTAGCCCTTTCGGGACGGCTCTATCGCCTAGCAGGCCTTATAAGGCACTTTCCCCCACATTCTATGAATATATTCGCG |
|  | Hap 12 | AAACATAGCGAATTACATCTTGTATTCTAGCCCTTTCGTGTCGGCTCTATCGCCTAGCAGACCTTATAAGGCACTTTCCCCCACATTCTATGAATATATTCGCG |
|  | Hap 13 | AAACATAGCGAATTACATCTTGTATTCTAGCCCTTTCGTGACGGCTCTATCGCCTAGCAGGCCTTATAAGGCACTTTCCCTCACATTCTATGAATATATTCGCG |
|  | Hap 14 | AAACATAGCGAATTACATCTTGTATTCTAGCCCTTTCGTGACGGCTCTATCGCCTAGCAGGCCTTATAAGGCACTTTCCCCCACATTCCATGAATATATTCGCG |
|  | Hap 15 | AAACATAGCGAATTACATCTTGTATTCTAGCCCTTTCGTGACGGCTCTATCGCCTAGCAGGCCTTATAAGGCACTTTCCCCCACATCCTATGAATATATTCGCG |
|  | Hap 16 | GAATGTGACGGACCACGTTCTGTATCTTAACCTTCCCATAATGGTTCCATCGCTCTGCCAGTTCTATAAAATACCTCCTCCTACATTCTGTAGACATATTCGTG |
|  | Hap 17 | AAACACGATCAACCACGTCCCACGTCTCGACCCTTCCATAATGGCTCCGTTACCTAGTAAGTCCCACAGGGCATCTCCCCCCGCACTTCGCAGACGCACTCGTT |
|  | Hap 18 | AAATGTGACGGACTACGTTCTGTATCTTAACCTTCCCATAATGGTTCCATCGCTCTGCCAGTTCTATAAAATACCTCCTCCTACATTCCGTAGACATATTCGTG |
|  | Hap 19 | AAATGTGACAGACTACGTTCTGTATCTTAACCTTCCCATAATGGTTCCATCGCTCTGCCAGTTCTATAAAATACCTCCTCCTACATTCCGTAGACATATTCGTG |
|  | Hap 20 | AAACCCGATCAGCCACGTCCCACATCTCGACCCTTCCATAATGACTTCGCTACCTAACAAGTCCCGCAGGGCATCTCCCTCCGCACTTCGCAGACGCACTCGTT |
|  | Hap 21 | AAACCCGATCAGCCACGTCCCACATCTCGACCCTTCCATAATGACTTCGCTACCTAACAAGTCCCACAGGGCATCTCCCTCCGCACTTCGCAGACGCGCTCGTT |
|  | Hap 22 | AAACACGATCAGCCACGTCCCACATCTCGACTCTTCCATAATGGCTCCGTTACCTAGCAAGTCCCACAGGGCATCTCCCCCCATACTTCGCAGACGCACTCGTT |
|  | Hap 23 | AGACCCGATCAGCCACGTCCCACATCTCGACCCTTCCATAATGACTTCGCTACCTAACAAGTCCCGCAGGGCATCTCCCTCCGCACTTCGCAGACGCACTCGTT |
|  | Hap 24 | AAACACGATCAGCCACGCCCCACATCTCGACTCTTCCATAATGGCTCCGTTACCTAGCAAGTCCCACAGGGCATCTCCCCCCATACTTCGCAGACGCACTCGTT |
|  | Hap 25 | AAACACGATCAGCCACATCCCACATCTCGACTCCTCCATAATGGCTCCGTTACCTAGCAAGTCCCACAGGGCATCTCCCCCCACACTTCGCAGACGCACCCGTT |
|  | Hap 26 | AAACACGATCAGCCACGTCCCACATCTCGACTCCTCCATAATGGCTCCGTTACCTAGCAAGTCCCACAGGGCATCTCCCCCCACACTTCGCAGACGCATCTGTT |
|  | Hap 27 | AAACATAGCAAATTACATCTTGTATTCTAGCCCTTTCGTGACGGCTCTATCGCCTAGCAGGCCTTATAAGGCACTTTCCCCCACATTCTATGAATATATTCGCG |
|  | Hap 28 | AAACATAGCGAATTACATCTTGTATTCTAGCCCTTTCGTGACGGCTCTATCGCCTAGCAGGCCTTATAAGGCACTTTCCCCCACATTCTATGAGTATATTCGCG |
|  | Hap 29 | AAACATAGCGAATTACATCTTGTATTCTAGTCCTTTCGTGACGGCTCTATCGCCTAGCAGGCCTTATAAGGCACTTTCCCCCACATTCTATGAATATATTCGCG |
|  | Hap 30 | AAACATAGCGAATTACATCTTGTATTCTAGCCCTTTCGTGACGGCTCTATCGCCTAGCAAGCCTTATAAGGCACTTTCCCCCACATTCTATGAATATATTCGCG |
|  | Hap 31 | AAACACGGCGAACCACGTCCCGCGTCTCGACCCTTCCATAATGGCTCCGTTACCTAGTAAGTCCCACAGGGCATCTCCCCCCGCACTTCGCAGACGCACTCGTT |
|  | Hap 32 | AAACATAGCGAATTATATCTTGTATTCTAGCCCTTTCGTGACGGCTCTATCGCCTAGCAGGCCTTATAAGGCACTTTCCCCCACATTCTATGAATATATTCGCG |
|  | Hap 33 | AAACATAGCGAATTACATCTTGTATTCTAGCCCTTTCGTGACGGCCCTATCGCCTAGCAGGCCTTATAAGGCACTTTCCCCCACATTCTATGAATATATTCGCG |
|  | Hap 34 | AAACATAGCGAATTACATCTTGTATTCTAGCCCTTTCGTGACGGCTCTATCGCCTAGCAGGCCTTATCAGGCACTTTCCCCCACATTCTATGAATATATTCGCG |
|  | Hap 35 | AAACATAGCGAATTACATCTTGTATTCTAGCCCTTTCGTGACGGCTCTATCGCCTAGCAGGCCTTATAAGGCGCTTTCCCCCACATTCTATGAATATATTCGCG |
|  | Hap 36 | AAGTATAACCGGCTACGTCCCACACCTCAACCCTTCTATAATGGCTCCATCACCTAGCAAATCCCACAAGGCACCCCTCCCCGCACTTTACAGACGCACTCGTT |
|  | Hap 37 | AAACATAGCGAATTACATCTTGTATTCTAGCCCTTTCGTGACGGCTCTATCGCCTAGCAGGCCTTATAAGGCACTTTCCCCCACATTCTATGAATATATTCACG |
| COII | Hap 1 | TCATCCTTACCACCACCCGATGGCCAATATCCATCCTTTAATGA |
|  | Hap 2 | CCATCCTCATTATCACCCGATGACTAATACCTATCTGTTAATGA |
|  | Hap 3 | CCATCCTCATTATCACCCGATGATTAATACCTATCTGTTAATGA |
|  | Hap 4 | CCGTCCTTATTATCACCCGATGACTGATACCTATCTGTTAACGA |
|  | Hap 5 | TCATTCCTACTACCATTAGGCAACTAACATCTGCCCTTTGATGA |
|  | Hap 6 | TCATCCTTACCACCACCCGATGACCAATATCCATCCTTTAATGA |
|  | Hap 7 | TCATTCTTACTACCATCAGGCGACTAATGTCTGCCCTTTGATGA |
|  | Hap 8 | TCATTCTTACTACCATCAGGCGACTAATGTCTGCCCTCTGATGA |
|  | Hap 9 | TCATCCTTATTATCACCCGATGACTAATACCTATCTGTTAATGA |
|  | Hap 10 | TCATCCTTATTATTACCCGATGACTAATACCTATCTGTTAATGA |
|  | Hap 11 | CCATCCTTATTATCACCCGATGACTAATACCTATCTGTTAATGA |
|  | Hap 12 | TCATCCTTATTATCATCCGATGACTAATACCTATCTGTTAATGA |
|  | Hap 13 | TCATCCTTATTATCACCCGATGACTAATACCTATCTGTTAGTGA |
|  | Hap 14 | CCATCCTTATTATCACCCGATGACTAGTACCTATCTGTTAATGA |
|  | Hap 15 | CCATCCTTATTATCACCCGATGACTAATACCTATCTGTTAATGG |
|  | Hap 16 | CCATCCTTATTATCGCCCGATGACTAGTACCTATCTGTTAATGA |
|  | Hap 17 | CCATCCTTATTATCACCCGATGACTGATACCTATCTGTTAACGA |
|  | Hap 18 | TTATCCTTACCACCACCCGATGGCCAATATCCATCCTTTAATGA |
|  | Hap 19 | TCATCCTTACCGCCACCCGATGGCCAATATCCATCCTTTAATGA |
|  | Hap 20 | CCACCCTCATTATCACCCGATGACTAATACCTATCTGTTAATGA |
|  | Hap 21 | TCATCCTTGCCACCACCCGATGGCCAATATCCATCCTTTAATGA |
|  | Hap 22 | TCATCCTTACCACCACCCGATGGCCAATATCCATCCTTTAATAA |
|  | Hap 23 | CCATCTTTATTATCACCCAATGACTAATACTTATTTGTTAATGG |
|  | Hap 24 | TCATCCTTACTACCACCCGATGGCCAATATCCATCCTTTAATGA |
|  | Hap 25 | TCATCCTTACCACCACCCGATGGCCAATATCCATCCTTCAATGA |
| Cytb | Hap 1 | CCTTGATCTCCTCCTCTTGGTAATTATCGACAGTTTCCATCCATTCACCCTATCGACTATAAGATGCACTTCAAGAT |
|  | Hap 2 | CCTTGATCTCCTCCTCTTGGTGATTATCGACAGTTTCCATCCATTCACCCTATCGACTATAAGACGCACTTCAAGAT |
|  | Hap 3 | CCTTGATCTCCTCCTCTTGGTAATTATCGACAGTTTCCATCCATTCACCCTATCGACTATAAGACGTACTTCAAGAT |
|  | Hap 4 | CCTTGATCTCCTCCTCTTGGTAATTATCGACAGTTTCCATCCATTCACCCTATCGACTATAAGACGCACTTCAAGAT |
|  | Hap 5 | CCTTGATCTCCTCCTCTTGGTAATTATCGACAGTTTCCATCCATTCACCCTATCGACTATGAGACGCACTTCAAGAT |
|  | Hap 6 | TCTTGACTCCCCCTCCTGGGCATCTATCGACGTACCTCACTTGTTTGTCCCGTCAACTACATGGCACATTTCTAGAC |
|  | Hap 7 | CCTTGATCTCCTCCTCTTGGTAATTATCGACAGTTTCCATCCATTCACCCTATCGACTATAAGACGCACTTCAAGAG |
|  | Hap 8 | CCTTGATCTCCTCCTCTTGGTAATTATCGACAGTTTCCATCCATTCACCCTATCGACCATAAGACACACTTCAAGAT |
|  | Hap 9 | TCTTGACTCCCCCTCCTGGACATCTATCGACGTACCTCACTTGTTTGTCCCGTCAACTACATGGCACATTTCTAGAC |
|  | Hap 10 | CCTTGACTCCCCCTCCTGGGCATCTATCGACGTACCTCACTTATTTGTCCCGTCAACTACATGGCACATTTCTAGAC |
|  | Hap 11 | TCTTGACTCCCCCTCCTGGGCATCTGTCGACGTACCTCGCTTGTTTGTCCCGTCAACTACATGGCACATTTCTAAAC |
|  | Hap 12 | CCCTAATCCCTTCCCCCTGGCACTTACTGTCATCTTCTACTCACCCACTCCGCTAATTGCATAACACATCTTTGGGC |
|  | Hap 13 | CCTTGATCTCCTCCTCTTGGTAATTATCGATAGTTTCCATCCATTCACCCTATCGACTATAAGACGCACTTCAAGAT |
|  | Hap 14 | CCTTGATCTCCTCCTCTTGGTAATTATCGACAGTTTCCATCCATTCACCCTATCGACTATAAGACGCACTGCAAGAT |
|  | Hap 15 | CCCTGATCCCTTCCCCTTGGCACTCATTGTCATCTTCTACTCACCCACTTCGCTAATTGCATAACACATCTTTAGGC |
|  | Hap 16 | CCCTGATCCCTTCCCCTTGGCACTCATTGTCATCTTCTACTCACCCACTTCGCTAATTGCATAACACCTCTTTAGGC |
|  | Hap 17 | CCTTGACTCCCCCTCCTGGGCATCTATCCACGTACCTCACTTGTTTGTCCCGTCAACTACATGGCACATTTCTAGAC |
|  | Hap 18 | CCTTGACTCCCCCTCCTGAGCATCTATCGACGTACCTCACTTATTTGTCCCGTCAACTACATGGCACATTTCTAGAC |
|  | Hap 19 | CCTTGATCTCCTCCTCTTGGTAATTATCGACAGTTTCCATCCATTCACCCTATCAACTATAAGACGCACTTCAAGAT |
|  | Hap 20 | TCTTGACTCCCCCTCCTGGGCATCTATCAACGTACCTCACTTGTTTGTCCCGTCAACTACATGGCACATTTCTAGAC |
|  | Hap 21 | CCTTGATCTCCTCCTCTTGGTAATTATCGACAGTTTCCATCCATTCACCCTATCGACTATAAGACGCACTTCAGGAT |
|  | Hap 22 | CCTTGATCTCCTCCTCTTGGTAATTATCGACAGTTTCCATCCATTCACCCTATCGACTATAAGACACACTTCAAGAT |
|  | Hap 23 | CTTGGGCTCTCCTTCCTGGGCATCTATCGACGTCTTTCACTTATTTACCCCGTCACCTACATAACACATTTCTAGAC |
|  | Hap 24 | CCTTGATCTCCTCCTTTTGGTAATTATCGACAGTTTCCATCCATTCACCCTATCGACTATAAGACGCACTTCAAGAT |
|  | Hap 25 | CCTTAATCTCCTCCTCTTGGTAATTATCGACAGTTTCCATCCATTCACCCTATCGACTATAAGACGCACTTCAAGAT |
|  | Hap26 | CCTTGATCTCCTCCTCTTGGTAATTATCGACAGTTTCCATCCATTCACCCTATCGACTATAAGGCGCACTTCAAGAT |

**Table S3.** Molecular diversity indices for eight regions mitochondrial COII and Cytb

| Gene | Index | Region (*Ns*) | | | | | | | | | Total (145) | | | |
| --- | --- | --- | --- | --- | --- | --- | --- | --- | --- | --- | --- | --- | --- | --- |
|  |  | A (36) | B (14) | C (24) | D (17) | E (23) | F (6) | G (3) | H (22) |  | | | |  |
| COII |  |  |  |  |  |  |  |  |  |  | |  |  |  |
|  | *Nh* | 5 | 2 | 4 | 5 | 3 | 3 | 1 | 10 | 25 | | | |  |
|  | *nTi*/*nTv* | 11 | 10 | 13 | 14 |  | 7.5 |  |  | 11.100 | | ± | 2.559 |  |
|  | π | 0.036 | 0.091 | 0.028 | 0.042 | 0.051 | 0.202 | 0 | 0.043 | 0.062 | | ± | 0.062 |  |
|  | *h* | 0.213 | 0.363 | 0.308 | 0.427 | 0.534 | 0.733 | 0 | 0.862 | 0.430 | | ± | 0.278 |  |
| Cytb |  |  |  |  |  |  |  |  |  |  | |  |  |  |
|  | *Nh* | 11 | 3 | 7 | 3 | 4 | 3 | 1 | 3 | 26 | | | |  |
|  | *nTi*/*nTv* | 6.6 | 4.1 | 5.3 | 4.5 |  | 8.5 |  | 2 | 5.184 | | ± | 2.226 |  |
|  | π | 0.057 | 0.167 | 0.045 | 0.050 | 0.025 | 0.261 | 0 | 0.015 | 0.078 | | ± | 0.090 |  |
|  | *h* | 0.484 | 0.473 | 0.605 | 0.228 | 0.549 | 0.733 | 0 | 0.567 | 0.455 | | ± | 0.233 |  |
| *Ns*, number of samples examined; *Nh*, number of haplotypes; *nTi*/*nTv*, the ratio of transitions to transversions; π, nucleotide diversity; *h*, haplotype diversity. | | | | | | | | | | | | | | |

**Table S4.** Analysis of molecular variance (AMOVA) for mitochondrial COII and Cytb of *V. emeryi*

| Gene | Source of variation | d.f.^z^ | Percentage variation |
| --- | --- | --- | --- |
| COII |  |  | |
|  | Among regions | 7 | 73.25 |
|  | Among populations within regions | 53 | 0 |
|  | Within populations | 84 | 31.28 |
|  | Total | 144 | 100.00 |
| Cytb |  |  | |
|  | Among regions | 7 | 75.90 |
|  | Among populations within regions | 53 | 4.37 |
|  | Within populations | 84 | 19.73 |
|  | Total | 144 | 100.00 |
| ^Z^ = degrees of freedom. | | | |

**Table S5.** Population pairwise *F*_ST_ values between regions for COII

|  | Region A | Region B | Region C | Region D | Region E | Region F | Region G | Region H |  |
| --- | --- | --- | --- | --- | --- | --- | --- | --- | --- |
| Region A | - |  |  |  |  |  |  |  |  |
| Region B | 0.09493* | - |  |  |  |  |  |  |  |
| Region C | -0.01847 | 0.08659 | - |  |  |  |  |  |  |
| Region D | -0.02087 | 0.03641 | -0.02562 | - |  |  |  |  |  |
| Region E | 0.85109** | 0.71718** | 0.85851** | 0.82528** | - |  |  |  |  |
| Region F | 0.75403** | 0.52726** | 0.75525** | 0.68389** | 0.68754** | - |  |  |  |
| Region G | 0.90241** | 0.75265** | 0.92441** | 0.89008** | 0.74567** | 0.61745 | - |  |  |
| Region H | 0.83323** | 0.67595** | 0.84591** | 0.81033** | 0.35705** | 0.65004** | 0.73727** | - |  |
| *P < 0.05; **P < 0.01 | | | | | | | | | |

**Table S6.** Population pairwise *F*_ST_ values between regions for Cytb

|  | Region A | Region B | Region C | Region D | Region E | Region F | Region G | Region H |  |
| --- | --- | --- | --- | --- | --- | --- | --- | --- | --- |
| Region A | - |  |  |  |  |  |  |  |  |
| Region B | 0.10020* | - |  |  |  |  |  |  |  |
| Region C | -0.02341 | 0.08826 | - |  |  |  |  |  |  |
| Region D | -0.03421 | 0.04885 | -0.04233 | - |  |  |  |  |  |
| Region E | 0.89522** | 0.76544** | 0.91509** | 0.91151** | - |  |  |  |  |
| Region F | 0.78450** | 0.53222** | 0.78777** | 0.74998** | 0.73979** | - |  |  |  |
| Region G | 0.88152** | 0.66421* | 0.90726** | 0.89781** | 0.87842** | 0.52574 | - |  |  |
| Region H | 0.90339** | 0.77843** | 0.92634** | 0.92507** | 0.18511** | 0.76359** | 0.92761** | - |  |
| *P < 0.05; **P < 0.01 | | | | | | | | | |

**Table S7.** Neutrality test for COII

|  | Region (*Ns*) | | | | | | | | Mean | ± | s.d. |
| --- | --- | --- | --- | --- | --- | --- | --- | --- | --- | --- | --- |
|  | A (36) | B (14) | C (24) | D (17) | E (23) | F (6) | G (3) | H (22) |  |  |  |
| Tajima’s *D* | -2.4887** | 0.6040 | -2.3420** | -2.2301** | 1.9452 | 1.1842 | - | -1.0531 | -0.5476 | ± | 1.7284 |
| Tau (τ) | 3.0 | 3.0 | 3.0 | 0.5 | 5.9 | 16.5 | - | 1.8 | 4.2102 | ± | 5.2764 |
| SSD | 0.0090 | 0.1817** | 0.0089 | 0.0138 | 0.1507** | 0.2202 | - | 0.0042 | 0.0736 | ± | 0.0936 |
| Raggedness index | 0.4899 | 0.6692 | 0.2787 | 0.1674 | 0.3028 | 0.2933 | - | 0.0577 | 0.2824 | ± | 0.2193 |
| **P < 0.01. *Ns*, number of samples; SSD, sum of squared deviation. | | | | | | | | | | | |

**Table S8.** Neutrality test for Cytb

|  | Region (*Ns*) | | | | | | | | Mean | ± | s.d. |
| --- | --- | --- | --- | --- | --- | --- | --- | --- | --- | --- | --- |
|  | A (36) | B (14) | C (24) | D (17) | E (23) | F (6) | G (3) | H (22) |  |  |  |
| Tajima’s *D* | -2.5060** | 0.5794 | -2.5296** | -2.4666** | 0.6303 | 1.3112 | - | 0.9646 | -0.5021 | ± | 1.6957 |
| Tau (τ) | 0.8 | 0.0 | 0.8 | 3.0 | 5.3 | 37.2 | - | 2.5 | 6.2041 | ± | 12.6709 |
| SSD | 0.0070 | 0.3451** | 0.0161 | 0.0164 | 0.1040 | 0.2214 | - | 0.1427 | 0.1066 | ± | 0.1248 |
| Raggedness index | 0.1080 | 0.3843 | 0.1338 | 0.4729 | 0.3162 | 0.2933 | - | 0.5214 | 0.2787 | ± | 0.1841 |
| **P < 0.01. *Ns*, number of samples; SSD, sum of squared deviation. | | | | | | | | | | | |


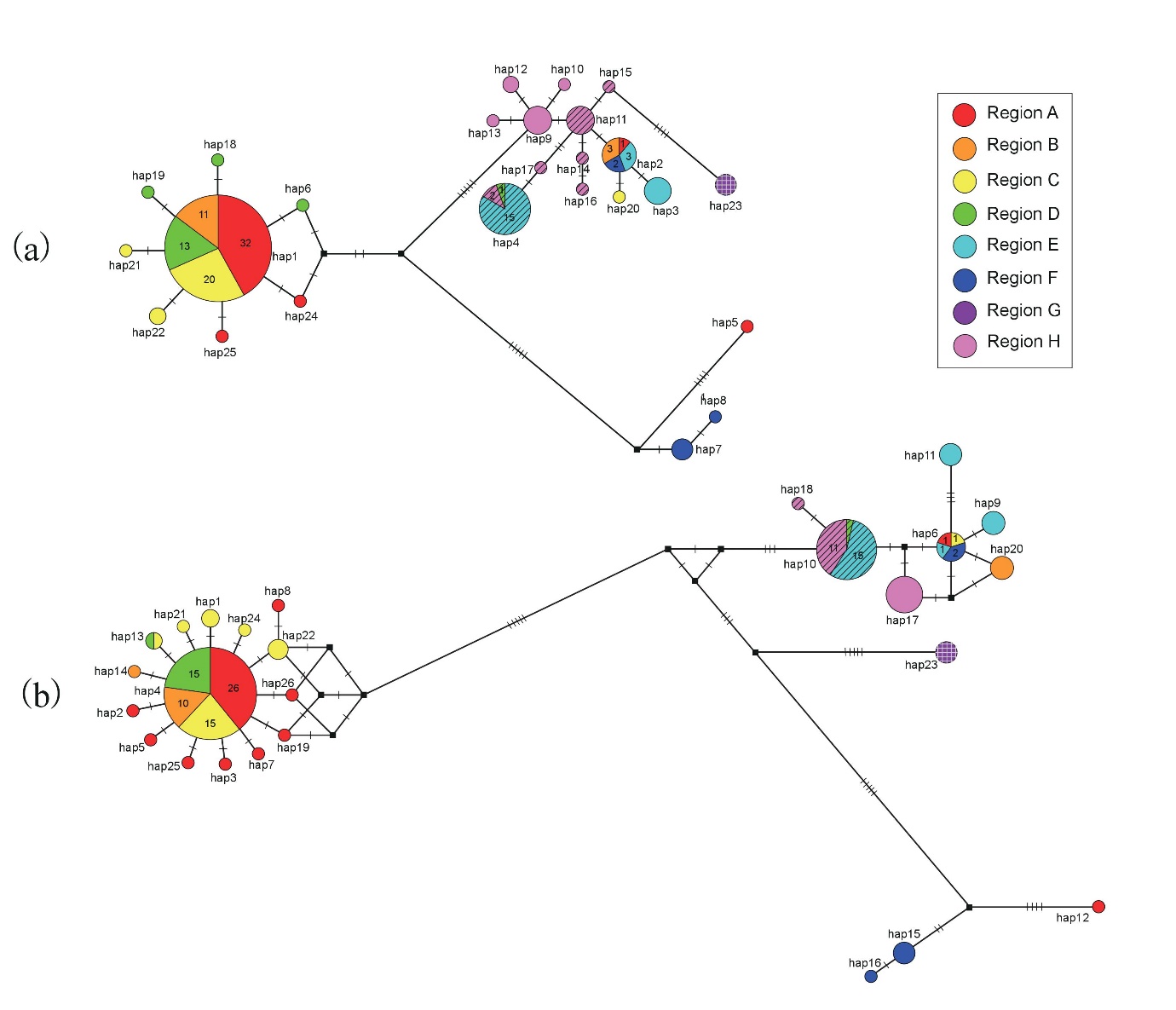


**Figure S1.** Haplotype network for COII (a) and Cytb (b). Circle size and line length are proportional to the haplotype frequency and the number of mutations, respectively. Geographic regions are color-coded. Numbers on the pie chart indicate the numbers of individuals with the particular haplotypes in the region. The wing morph is represented by the following pattern: solid, L morph; diagonal stripe, S morph; crossed stripe, wing type unknown. Black square dots represent potentially missing haplotypes. The number of short vertical lines represents the number of mutational steps. Five short vertical lines indicate more than five mutational steps.
